# Systematic evaluation of RNA-Seq preparation protocol performance

**DOI:** 10.1186/s12864-019-5953-1

**Published:** 2019-07-11

**Authors:** Hsueh-Ping Chao, Yueping Chen, Yoko Takata, Mary W. Tomida, Kevin Lin, Jason S. Kirk, Melissa S. Simper, Carol D. Mikulec, Joyce E. Rundhaug, Susan M. Fischer, Taiping Chen, Dean G. Tang, Yue Lu, Jianjun Shen

**Affiliations:** 1 Department of Epigenetics and Molecular Carcinogenesis, The University of Texas MD Anderson Cancer Center, Science Park, Smithville, TX 78957 USA; 2 Program in Genetics and Epigenetics, The University of Texas MD Anderson Cancer Center UTHealth Graduate School of Biomedical Sciences, The University of Texas MD Anderson Cancer Center, Smithville, TX 78957 USA; 3 Department of Pharmacology and Therapeutics, Roswell Park Cancer Institute, Buffalo, NY 14263 USA

**Keywords:** Next generation sequencing, RNA-Seq, Quality control

## Abstract

**Background:**

RNA-Seq is currently the most widely used tool to analyze whole-transcriptome profiles. There are numerous commercial kits available to facilitate preparing RNA-Seq libraries; however, it is still not clear how some of these kits perform in terms of: 1) ribosomal RNA removal; 2) read coverage or recovery of exonic vs. intronic sequences; 3) identification of differentially expressed genes (DEGs); and 4) detection of long non-coding RNA (lncRNA). In RNA-Seq analysis, understanding the strengths and limitations of commonly used RNA-Seq library preparation protocols is important, as this technology remains costly and time-consuming.

**Results:**

In this study, we present a comprehensive evaluation of four RNA-Seq kits. We used three standard input protocols: Illumina TruSeq Stranded Total RNA and mRNA kits, a modified NuGEN Ovation v2 kit, and the TaKaRa SMARTer Ultra Low RNA Kit v3. Our evaluation of these kits included quality control measures such as overall reproducibility, 5′ and 3′ end-bias, and the identification of DEGs, lncRNAs, and alternatively spliced transcripts. Overall, we found that the two Illumina kits were most similar in terms of recovering DEGs, and the Illumina, modified NuGEN, and TaKaRa kits allowed identification of a similar set of DEGs. However, we also discovered that the Illumina, NuGEN and TaKaRa kits each enriched for different sets of genes.

**Conclusions:**

At the manufacturers’ recommended input RNA levels, all the RNA-Seq library preparation protocols evaluated were suitable for distinguishing between experimental groups, and the TruSeq Stranded mRNA kit was universally applicable to studies focusing on protein-coding gene profiles. The TruSeq protocols tended to capture genes with higher expression and GC content, whereas the modified NuGEN protocol tended to capture longer genes. The SMARTer Ultra Low RNA Kit may be a good choice at the low RNA input level, although it was inferior to the TruSeq mRNA kit at standard input level in terms of rRNA removal, exonic mapping rates and recovered DEGs. Therefore, the choice of RNA-Seq library preparation kit can profoundly affect data outcomes. Consequently, it is a pivotal parameter to consider when designing an RNA-Seq experiment.

**Electronic supplementary material:**

The online version of this article (10.1186/s12864-019-5953-1) contains supplementary material, which is available to authorized users.

## Background

Omics technology, driven by next-generation sequencing (NGS) coupled with new and increasingly robust bioinformatics pipelines, has triggered exponential growth in the accumulation of large biological datasets. The first NGS study, published in 2005 [1], reported the highly accurate sequencing of 25 million DNA bases in less than a day, representing a vast improvement in cost and throughput over traditional Sanger sequencing methods. Shortly thereafter, NGS technology was applied to RNA sequencing (RNA-Seq) [2–5], and since then, the sensitivity, accuracy, reproducibility, and flexibility of RNA-Seq have made it the gold standard in transcriptomic research. Over the last ten years, approximately 53,700 RNA-Seq datasets have been deposited in the Gene Expression Omnibus (GEO) database [6]. These RNA-Seq datasets provide information about the whole transcriptome, including gene fusions, differential expression of coding and non-coding genes, and splice variants in different experimental conditions. Increasing evidence confirms that changes in the transcriptome are a result of biological alterations, making RNA-Seq a driving force behind the exploration of global regulatory networks in cells, tissues, organisms, and diseases.

RNA-Seq is used primarily to identify differentially expressed genes (DEGs) in different biological conditions, but it is also used to discover non-coding RNAs such as microRNAs and long non-coding RNAs (lncRNAs) [7]. RNA-Seq studies have already shown that differences in RNA preparation and enrichment during library preparation can cause fundamental variations in experimental outcomes. Hence, comprehensive evaluation of RNA-Seq library preparation methods by using different kits has provided a baseline from which to compare their overall capabilities and to guide future research applications. Several earlier studies have already identified potential confounding factors affecting RNA-Seq performance and analysis [8–15]. These include two large-scale projects--the Sequencing Quality Control project of the SEQC/MAQC-III (MicroArray Quality Control) Consortium, led by US Food and Drug Administration [8] and the Association of Biomolecular Resource Facilities (ABRF) next-generation sequencing (NGS) study [9], and other studies including the evaluation of three Illumina RNA-Seq protocols for degraded and low quantity samples [10], a study of gene qualification on clinical samples using Illumina TruSeq Stranded Total RNA and mRNA RNA-Seq protocols [11] and additional investigations focused on low-input or single-cell sequencing [12–15].

The SEQC project evaluated the sensitivity, specificity, reproducibility, and complexity of gene expression, DEGs, and splice junction detection from RNA-Seq performed at multiple sites, using the same commercial reference library and External RNA Controls Consortium (ERCC) RNA spike-in controls as well as experimental samples, but using different sequencing platforms and bioinformatics pipelines [8]. Overall, the SEQC project found that RNA-Seq data generated from vendor-prepared libraries were stable across sites but variable across protocols, implying that data variability likely originated from differences in library preparation and/or sequencing platforms. Parameters affecting library preparation include fragmentation time, ribosomal RNA (rRNA) depletion methods, cDNA synthesis procedures, library purification methods, ligation efficiency, and RNA quality. This study [8] also illustrated that for the most highly expressed genes, DEGs were consistently identified across sites and platforms and that de novo splice junction discovery was robust but sensitive to sequencing depth.

The ABRF-NGS study evaluated not only the sensitivity, specificity, reproducibility, and complexity of gene expression, but also differential gene expression and splice junction detection among different combinations of sequencing platforms and library preparation methods, taking into account size-specific fractionation and RNA integrity [9]. In general, the results across platforms and library preparation methods were highly correlated, but greater read depth was necessary to recover rare transcripts and splice site junctions present at low frequency, especially those resulting from putative novel and complex splicing events. Library preparation influenced the detection of non-polyA tail transcripts, 3′ UTRs, and introns, primarily due to inherent differences between rRNA reduction methods, i.e., rRNA depletion and polyA enrichment, with the former method capturing more structural and non-coding RNAs, and the latter method capturing more full-length mRNAs [9]. More importantly, although gene quantification was robust, transcriptome coverage was sensitive to the pipelines applied during the analyses; however, surrogate variable analysis proved useful in making direct comparisons across platforms.

Schuierer S. et al. [10] evaluated three Illumina library preparation kits, representing polyA selection, ribosomal RNA depletion and exon capture methods, respectively, on RNA-Seq samples in a wide range of input quantity and quality. They found ribosomal RNA depletion method had generally good performance whereas exon capture method performed the best for highly degraded RNA samples. Zhao S. et al. [11] evaluated polyA selection vs. rRNA depletion using clinical samples and recommended the former over the latter in most cases where the interest is protein-coding gene quantification.

More recently, increasing interest in investigating rare cell populations and detailed biological mechanisms has led to a demand for protocols generating high quality libraries from nanogram quantities of total RNA [12, 13] and even single cells [14, 15]. Dissecting the characteristics of RNA-Seq protocols designed to obtain data from low-input or degraded samples will benefit studies involving both rare cell populations and fixed clinical samples. For low-quantity RNA analysis, it has been established that the NuGEN protocol yields data with better transcriptome complexity but has less effective rRNA depletion, while the SMARTer Ultra Low RNA Kit has better performance on transcriptome annotation but demonstrates bias with respect to underrepresenting transcripts with high GC content [12]. cDNA amplification can help compensate for extremely small amounts of starting materials in low quantity RNA-Seq, but amplification itself may introduce problems, such as duplication, that affect library performance [12]. ABRF evaluated several low-input RNA amplification kits and identified certain underlying differences, such as two distinct categories of genes recovered in the libraries prepared with two distinct rRNA-reduction techniques, polyA enrichment and rRNA-depletion [13]. The sensitivity of gene detection and accuracy of gene expression level assessments were consistent across approaches but divergent across RNA input amounts. The SMARTer protocol provided a near perfect correlation between obtained values and the actual amount of ERCC standard included as a spike-in control [13]. Although this prior study provides insight into the effects of RNA amplification, it employed an artificial system using commercial RNA from TaKaRa mixed with the ERCC control RNAs, which likely oversimplifies the transcriptome complexity of real cells, thus necessitating similar work in whole-cell systems.

The source of data variation among different library preparation methods remains unclear. Therefore, in the present study, we carefully compared the results we obtained from several commercial RNA-Seq library preparation kits with different rRNA depletion and cDNA synthesis methods to understand the strength of each protocol. The first goal of our study was to investigate confounding factors in RNA-Seq library preparation protocols using three standard input kits: the TruSeq Stranded Total RNA and mRNA Library Prep Kits from Illumina, and a modified NuGEN Ovation® RNA-Seq System. Defining the properties of the data generated using these protocols may aid users in designing their future RNA-Seq strategies. The second part of our study was to thoroughly evaluate the SMARTer Ultra Low RNA Kit using mouse embryonic stem cells (mESCs). Our results demonstrated that the TruSeq Stranded mRNA protocol was the best for transcriptome profiling and that the TruSeq Stranded Total RNA and mRNA protocols were comparable, whereas the modified NuGEN protocol performed less well for whole transcriptome analysis, but might be a better choice for studies focused on non-coding RNAs. Lastly, although the results obtained with the SMARTer Ultra Low RNA Kit were comparable to those of the TruSeq Stranded mRNA kit for most metrics and for identification of DEGs, the absolute expression levels were only moderately correlated. We conclude that each RNA-Seq protocol has individual strengths for particular individual applications that need to be considered for a successful RNA-Seq experiment.

## Results

### Experimental design and RNA-Seq data quality metrics

**Fig. 1:**
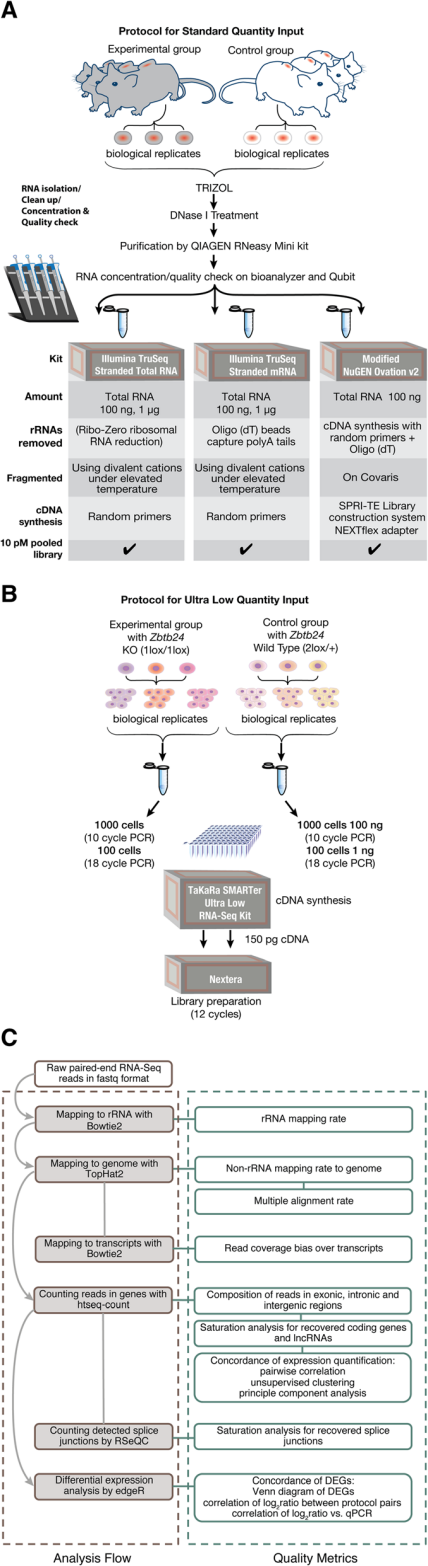
Experimental design and RNA-Seq data quality metrics. **a** Flow chart outlining the experimental design for comparing the three standard input RNA-Seq library preparation protocols. Six xenograft tumors, 3 from the control group and 3 from the experimental group, were used for all three protocols. Similar amounts of tumor tissue from control and experimental groups were used to isolate total RNA. Separate Illumina Stranded Total RNA and mRNA libraries were prepared using 100 ng and 1 μg RNA. The modified NuGEN Ovation v2 protocol library was prepared with 100 ng RNA. Images of the mice and vials were created by the Research Graphics department at MD Anderson Science Park (©MD Anderson), and the pipettes were taken from https://all-free-download.com/free-vectors/ **b** Flow chart outlining the ultra-low input protocol. Cells from 3 independently derived *Zbtb24* wild-type (2lox/+) mESC control lines and 3 independently derived *Zbtb24* knockout (1lox/1lox) mESC experimental lines were lysed directly in reaction buffer without isolating total RNA. One hundred cells (~ 1 ng RNA, 18 PCR cycles) and 1000 cells (~ 10 ng RNA, 10 PCR cycles) were used to make cDNA for the TaKaRa SMARTer Low Input RNA-Seq kit v3 protocol. One hundred-fifty pg of TaKaRa SMARTer-generated cDNA was then used to prepare the Nextera libraries. **c** A diagram depicting the data analysis flow and the data quality metrics used in this study to evaluate RNA-Seq protocols. The analysis steps are on the left and the data quality metrics that were derived from each analysis step are on the right

Figure 1 outlines the experimental design we used for testing the three standard input protocols (Illumina TruSeq Stranded Total RNA, Illumina TruSeq Stranded mRNA, and modified NuGEN Ovation v2) (Fig. 1a), the ultra-low input protocol (TaKaRa SMARTer Ultra Low RNA Kit) (Fig. 1b), the data analysis flow, and data quality evaluation metrics (Fig. 1c). The RNA-Seq datasets used in the current study were generated during two research-based projects. The first study assessed six xenograft tumors, three from the control group (biological replicates) and three from the experimental group (biological replicates) to test all three standard input protocols (Fig. 1a). Because one of the xenograft tumors from the control group was used up, a different tumor (from a different mouse) had to be used for the libraries prepared with the TruSeq Total RNA protocol (100 ng) and the TruSeq mRNA protocol (100 ng). The second study assessed three mESC cell lines (biological replicates) from *Zbtb24* knockout (1lox/1lox) clones compared with three wild-type (2lox/+) clones (biological replicates) using the TaKaRa SMARTer Ultra Low RNA protocol directly on cells with no RNA preparation step. When RNA was isolated, all total RNA samples had RNA integrity (RIN) numbers > 8.90.

We used the manufacturer-recommended optimal input amounts (1 μg for both the Illumina TruSeq Stranded Total RNA and the Illumina TruSeq Stranded mRNA protocols; and 100 ng for the modified NuGEN Ovation v2; hereafter, “standard protocol”) (Fig. 1a). In addition, we also compared all three of these protocols with 100 ng input RNA (Fig. 1a and in the Additional file Figures). As described in a recent study, and as shown in Fig. 1a, the Illumina TruSeq Stranded Total RNA protocol uses Ribo-Zero to remove rRNA, whereas the TruSeq Stranded mRNA protocol enriches mRNA through polyA selection [11]. In contrast, as shown in Fig. 1a, the modified NuGEN Ovation v2 protocol synthesizes cDNA directly from total RNA with a combination of random primers and oligo [15], and followed by cDNA fragmentation on Covaris. On the other hand, both TruSeq protocols use divalent cations under elevated temperature to fragment purified RNAs. For the TaKaRa SMARTer Ultra Low RNA Kit, we used total RNA from 100 mESCs cells and 1000 mESCs cells or approximately 1 and 10 ng RNA, respectively. To check whether this modified ultra-low input protocol was capable of generating quality data, we compared the mESC dataset derived from the TaKaRa SMARTer cDNA synthesis step combined with Nextera library preparation, to the high-quality datasets obtained using the TruSeq Stranded mRNA protocol with 2 μg total RNA as the input level.

The data analysis flow and the data quality metrics used in this study to evaluate RNA-Seq protocols are diagrammed in Fig. 1c and detailed below.

### Mapping statistics (standard input protocols)

**Fig. 2:**
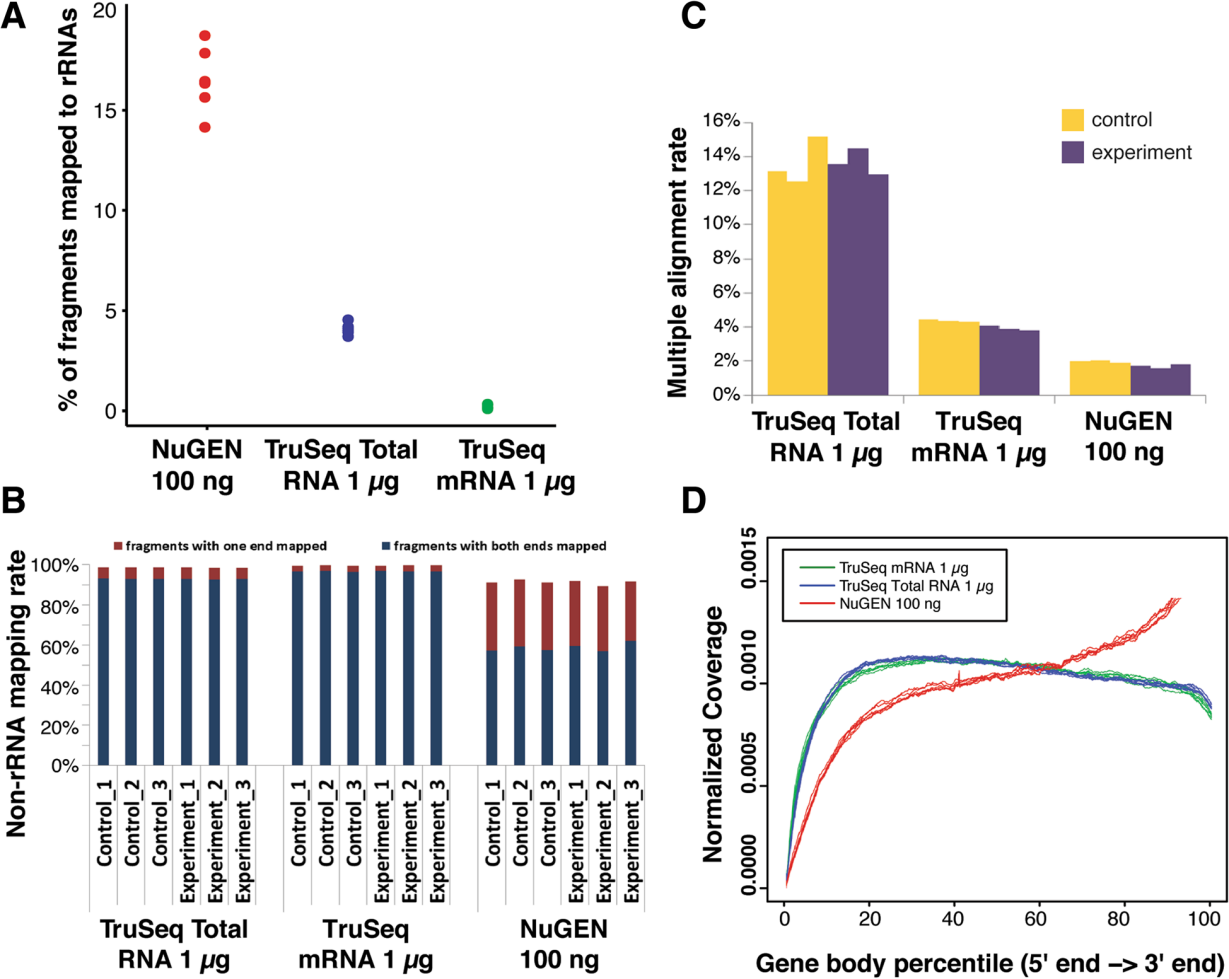
Mapping statistics and read coverage over transcripts for all the libraries prepared with standard input protocols. **a** The rRNA mapping rate was calculated as the percentage of fragments that were mappable to rRNA sequences. **b** The non-rRNA mapping rate was calculated from all the non-rRNA fragments as the percentage of fragments with both ends or one end mapped to the genome. **c** Multiple alignment rates were determined from non-rRNA fragments that were mapped to multiple locations of the genome. **d** Read-bias was assessed using the read coverage over transcripts. Each transcript was subdivided evenly into 1000 bins and the read coverage was averaged over all the transcripts

The high abundance of rRNA in cells creates an important problem in RNA-Seq experiments. rRNA contamination of samples wastes reagents and decreases the recovery of other RNA species of interest. Therefore, we wanted to determine the efficacy of each protocol in removing rRNA. We found that for the libraries created with the modified NuGEN, TruSeq Stranded Total RNA, and TruSeq Stranded mRNA protocols, ~ 17, 5, and 1% of fragments, respectively, could be mapped to rRNA genes (Fig. 2a and Additional file 1: Figure S1A), indicating that in our conditions, the modified NuGEN protocol was inferior to the other two protocols in reducing rRNA contamination. After removing the rRNA reads, we mapped the remaining reads to the whole mouse genome using TopHat. The percentages of fragments with at least one end mapped to the genome were ~ 98% for both TruSeq protocols, and ~ 90% for the modified NuGEN protocol (Fig. 2b and Additional file 1: Figure S1B). The percentages of fragments with both ends mapped were > 93%, for both TruSeq Stranded Total RNA and TruSeq Stranded mRNA libraries, and ~ 60% for the modified NuGEN library (Fig. 2b and Additional file 1: Figure S1B). The percentages of fragments mapped to multiple locations of the genome accounted for ~ 12–20%, ~ 3–5%, and ~ 2% of total non-rRNA fragments from the samples prepared with the TruSeq Stranded Total RNA, TruSeq Stranded mRNA, and modified NuGEN protocols, respectively (Fig. 2c and Additional file 1: Figure S1C).

### Read coverage over transcripts (standard input protocols)

Positional signal bias in RNA-Seq data can lead to inaccurate transcript quantification. Therefore, we examined the read coverage over transcripts longer than 1000 bps and found excessive enrichment of fragments at the 3′-end and depletion of signal at the 5′-end for samples prepared with the modified NuGEN protocol (Fig. 2d and Additional file 1: Figure S1D). Reads from the TruSeq Stranded Total RNA and TruSeq Stranded mRNA protocols were more evenly distributed along the entire length of the transcript (Fig. 2d and Additional file 1: Figure S1D). Closer examination of each nucleotide within 1000 bps of the 5′- and 3′- ends confirmed that the modified NuGEN protocol failed to capture the RNA signal towards the 5′-end (Additional file 2: Figure S2A, C), and also suggested that the TruSeq Stranded mRNA protocol missed the signal within 200 bp of the 3′-end, compared to the TruSeq Stranded Total RNA protocol (Additional file 2: Figure S2B, D).

### Representation of the transcriptome (standard input protocols)

**Fig. 3:**
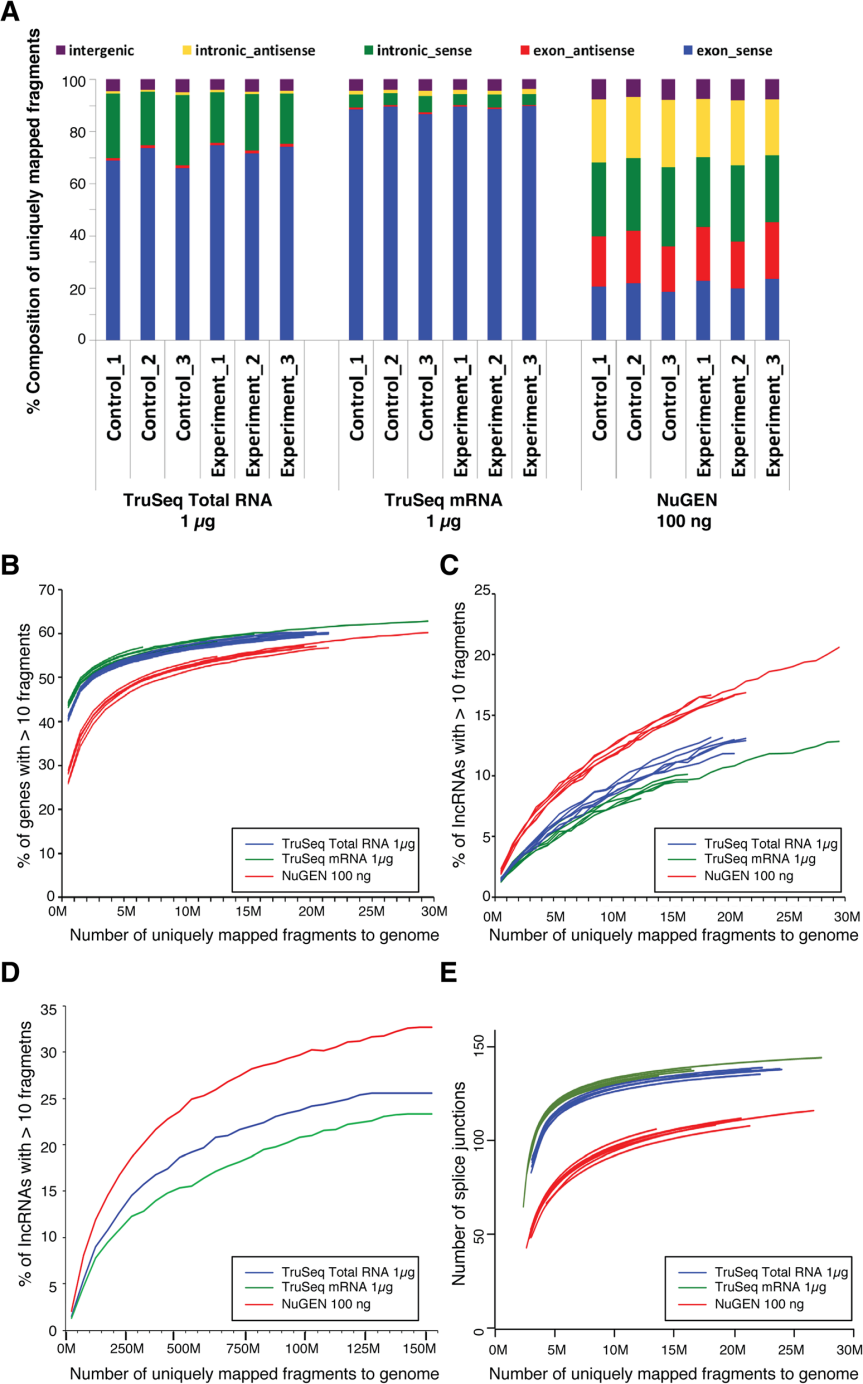
Representation of the transcriptome for all the libraries prepared with standard protocols. **a** Composition of the uniquely mapped fragments, shown as the percentage of fragments in exonic, intronic, and intergenic regions. According to the direction of transcription, exonic and intronic regions were further divided into sense and antisense. **b** Saturation analysis showing the percentage of coding genes recovered (calculated as the genes with more than 10 fragments) at increasing sequencing depth. **c**-**d** Saturation analysis showing the percentage of lncRNAs recovered (calculated as the lncRNAs with more than 10 fragments) at increasing sequencing depth. In C, the six libraries created using each of three protocols (18 libraries total) are plotted individually. In D, the six libraries from the same protocol were pooled. **e** Saturation analysis showing the number of splice junctions recovered at increasing sequencing depth

To assess how well the entire transcriptome was represented within the libraries generated by the three RNA-Seq protocols, we first investigated the composition of uniquely mapped fragments in exonic, intronic, and intergenic regions (Fig. 3a and Additional file 3: Figure S3A). We found that for the TruSeq Stranded Total RNA and mRNA protocols, respectively, approximately 67–84% and 88–91% of the fragments were from exonic regions; 14–28 and < 10% were from intronic regions; and the remaining 3–5% were from intergenic regions. For the modified NuGEN protocol, only 35–45% of the fragments were from exonic regions; 47–56% were from intronic regions; and less than 10% were from intergenic regions. Since only the TruSeq protocols are strand-specific, as expected, the majority of the fragments in exonic and intronic regions were from the sense strand of the genes, whereas for the NuGEN libraries about half of the fragments were from the sense strand and the other half were from the antisense strand of the genes.

To evaluate the capability of the RNA-Seq protocols for detecting coding genes and lncRNAs, we performed saturation analysis to count the number of coding genes and lncRNAs detected at increasing sequencing depth. For coding genes, the saturation curves from the TruSeq Stranded Total RNA and mRNA libraries looked very similar and were superior to those from the NuGEN libraries (Fig. 3b and Additional file 3: Figure S3B). For lncRNAs, the modified NuGEN protocol outperformed both the TruSeq Stranded Total RNA and mRNA protocols, yielding more lncRNAs at the same sequencing depth (Fig. 3c Additional file 3: Figure S3C). However, for lncRNAs, none of the libraries were close to saturation at the sequencing depth used for our experiments. To examine the sequencing depth required to reach saturation for lncRNA detection, we repeated our saturation analysis after pooling samples from the same RNA-Seq protocol together. Our analysis showed that the modified NuGEN protocol still exceeded the other two protocols in lncRNA recovery, even when sequencing depth approached saturation (Fig. 3d and Additional file 3: Figure S3D).

Another important application of RNA-Seq is to identify alternatively spliced variants, which frequently occur in mammalian genes [16]. In this regard, we conducted saturation analysis comparing the number of reads to the number of detected splice sites (Fig. 3e and Additional file 3: Figure S3E). We recovered the lowest number of splice junctions using the modified NuGEN protocol and the highest number with the TruSeq Stranded mRNA protocol.

### Concordance of expression quantification (standard input protocols)

**Fig. 4:**
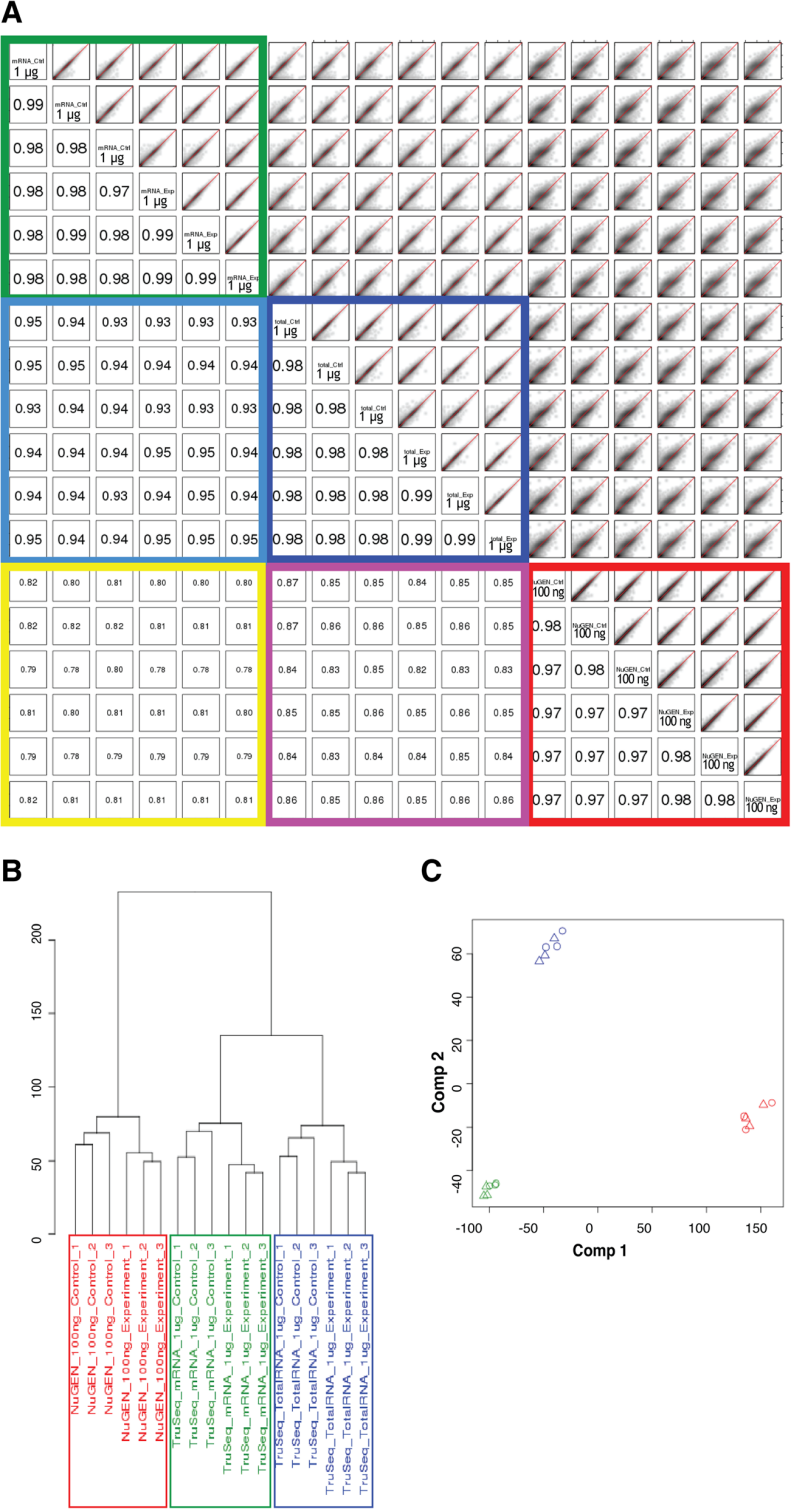
Concordance of expression quantification between the libraries prepared with standard input protocols. **a** Scatter plots in a smoothed color density representation (top-right panel) and Spearman’s rank correlation coefficients (bottom-left panel) for all pairs of libraries using log_2_(cpm + 1) values. **b** Unsupervised clustering of all the libraries using log_2_(cpm + 1) values. Euclidean distance with complete linkage was used to cluster the libraries. **c** Principal component analysis (PCA) of all the libraries, using log_2_(cpm + 1) values. The values for each gene across all the libraries were centered to zero and scaled to have unit variance before being analyzed. Circles and triangles represent control and experimental libraries, respectively (NuGEN, red; TruSeq mRNA, green; TrueSeq Total RNA, blue). For all analyses in Fig. 4, genes represented by fewer than 10 fragments in all the libraries were excluded

Spearman’s rank correlation coefficients between samples based on count per million (cpm) fragments mapped to exons values were calculated to assess the concordance of the three standard RNA-Seq protocols on expression quantification. The correlation coefficients were greater than 0.97 between samples prepared using the same protocol, regardless of whether the samples were biological replicates of the same condition or from different conditions. The correlation coefficients between samples prepared using different protocols were lower: 0.93–0.97 between the TruSeq Stranded Total RNA and mRNA protocols, 0.80–0.87 between the TruSeq Stranded Total RNA and modified NuGEN protocols, and 0.77–0.82 between the TruSeq Stranded mRNA and modified NuGEN protocols (Fig. 4a and Additional file 4: Figure S4A). Unsupervised clustering demonstrated that the whole transcriptome expression profiles obtained from TruSeq Stranded Total RNA and mRNA libraries were more similar to each other than either was to the NuGEN libraries (Fig. 4b and Additional file 4: Figure S4B). Principal component analysis (PCA) recapitulated the clustering analysis: the NuGEN libraries were separated from the TruSeq libraries in the first component, whereas the TruSeq Stranded Total RNA and mRNA libraries were separated in the second component (Fig. 4c and Additional file 4: Figure S4C). Further investigation revealed the TruSeq protocols tended to capture genes with higher expression and GC content, whereas the modified NuGEN protocol tended to capture longer genes (Additional file 7: Figure S7B-C). Comparing the TruSeq mRNA protocol to the TruSeq Total RNA protocol, showed that the TruSeq mRNA protocol preferentially recovered genes with higher GC content and shorter length (Additional file 7: Figure S7A). To exclude the possibility that these differences stemmed from batch effects, such as different set of libraries being prepared at different times, we included additional technical replicates, prepared at different times, for the TruSeq Stranded Total RNA and mRNA protocols (1 μg). Unsupervised clustering suggested that the distance between technical replicates of the same protocol was closer than the distance between samples prepared with different protocols (Additional file 5: Figure S5A). The technical replicate libraries generated using the same protocol clustered together and were separated from those of different protocols in PCA (Additional file 5: Figure S5B). Taken together, these results demonstrate that the variability among these library preparation protocols was not primarily due to batch effects.

### Concordance of DEGs recovered with standard input protocols

**Fig. 5:**
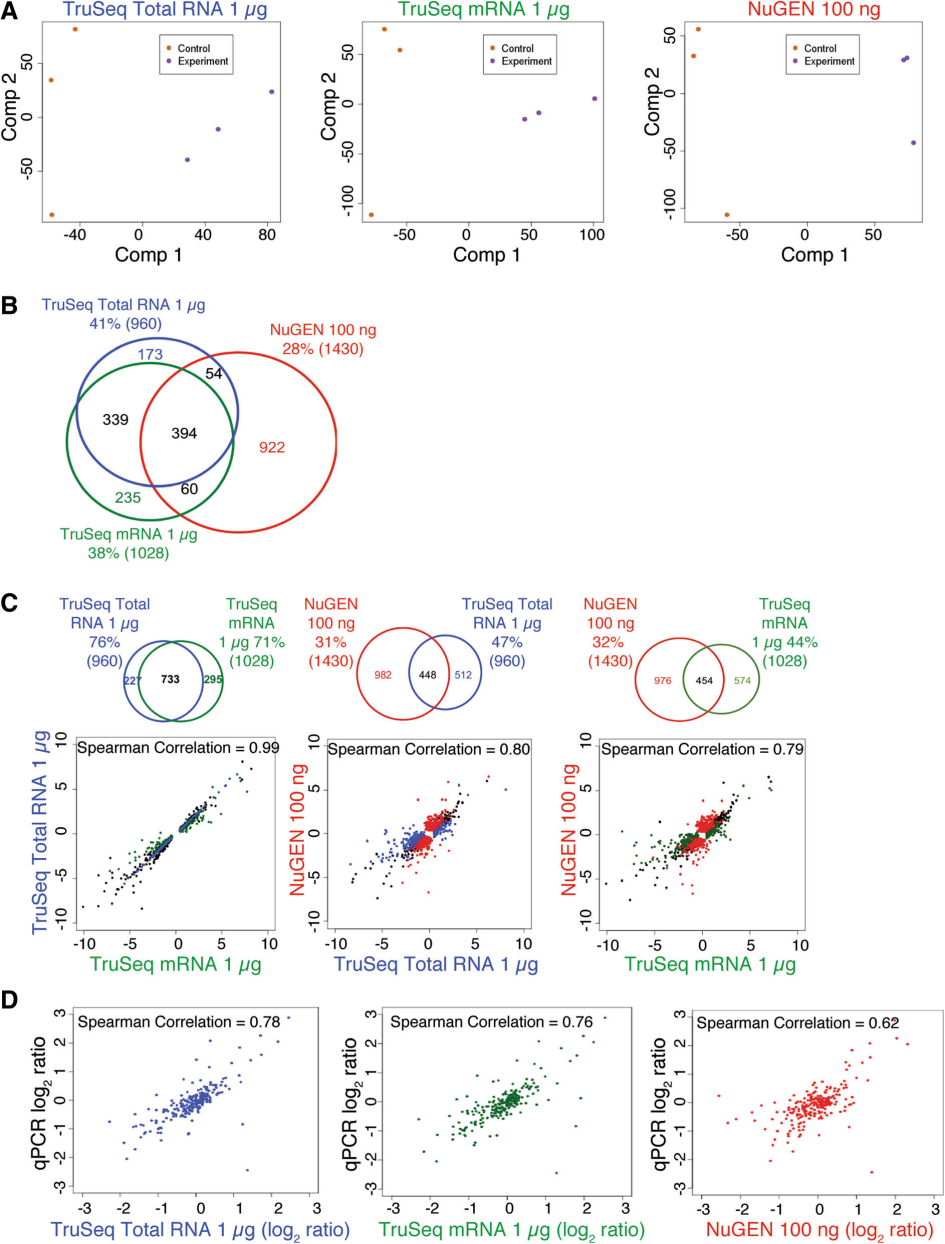
Concordance of differentially expressed genes (DEGs) recovered from libraries prepared with standard protocols. **a** Principle component analysis (PCA) was performed on the libraries prepared with each standard protocol. **b** Venn diagram showing the number of DEGs recovered with the three standard protocols. **c** Pairwise scatter plots of log_2_ ratio values comparing the DEGs identified in the tumor tissues of control and experimental mice. The black dots represent genes that were called as differentially expressed in libraries from both protocols, colored dots represent genes that were called as differentially expressed in the libraries from only one protocol. The Spearman’s rank correlation coefficient is shown at the top of each plot. The Venn diagram above each plot shows the number of DEGs recovered with the specified protocols. **d** Scatter plots of log_2_ ratio values calculated between tumor tissues of control and experimental mice for each protocol vs. qPCR. Spearman’s rank correlation coefficient is shown at the top of each plot

PCA demonstrated that all protocols could distinguish between samples representing different biological conditions (Fig. 5a and Additional file 6: Figure S6A). Three hundred ninety-four DEGs were detected across all three RNA-Seq library preparation protocols, accounting for 41, 38, and 28% of the total DEGs detected when using the TruSeq Stranded Total RNA, TruSeq Stranded mRNA, and modified NuGEN protocols, respectively (Fig. 5b). The pairwise scatter plots of log_2_ ratio values between DEGs from control and experimental mouse tumor tissues showed that the TruSeq Stranded Total RNA and mRNA results were more highly correlated with each other (Spearman’s correlation coefficient = 0.99) than either was with the modified NuGEN protocol (Spearman’s correlation coefficient = 0.80 and 0.79, respectively) (Fig. 5c and Additional file 6: Figure S6B). That is, the TruSeq Total RNA and mRNA protocols yielded more shared DEGs than either did with the modified NuGEN protocol (Fig. 5c and Additional file 6: Figure S6B). To evaluate how accurate the DEG calls were, we performed qPCR for 288 genes that RNA-Seq data indicated were differentially expressed, and compared the log_2_ ratio values for these genes as derived from the various RNA-Seq library preparation protocols and qPCR (manuscript in preparation). The DEGs recovered with the TruSeq Total RNA and mRNA protocols had correlation coefficients of 0.78 and 0.76 vs. qPCR, whereas the modified NuGEN protocol had a correlation coefficient of 0.62 (Fig. 5d). In short, the libraries produced by all three standard protocols were sufficient to detect DEGs. However, independent validation of DEGs by qPCR indicated that the differential expression results from the TruSeq Stranded Total RNA and mRNA protocols might be more accurate than those from the modified NuGEN protocol.

### Mapping statistics, read coverage bias and transcriptome representation (ultra-low protocol)

**Fig. 6:**
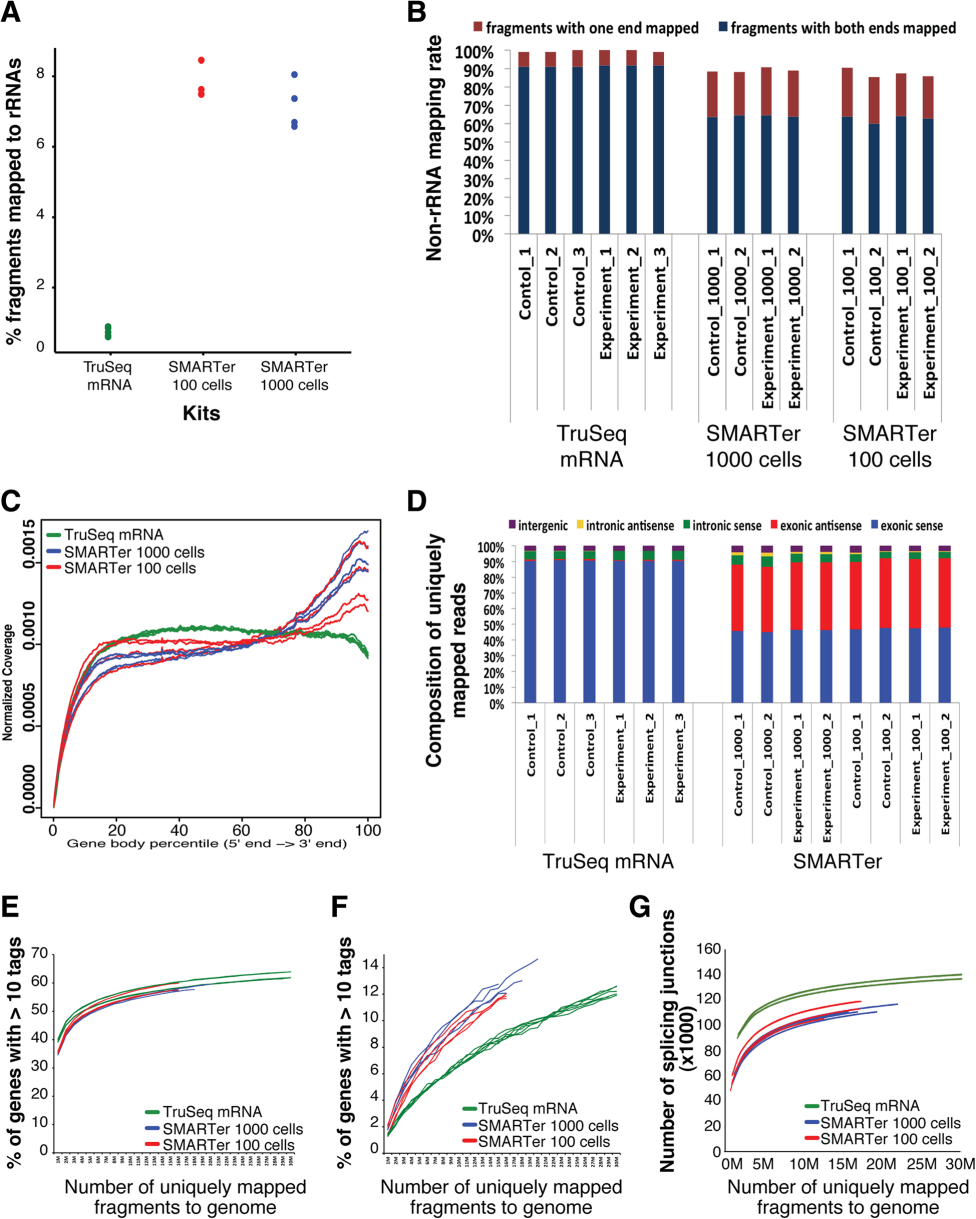
Mapping statistics, read coverage bias, and transcriptome representation for libraries prepared using the SMARTer Ultra Low RNA Kit. **a** The percentage of fragments mapped to rRNA sequences. **b** Of all the non-rRNA fragments, the percentage of fragments with both ends or one end mapped to the genome. **c** The read coverage over transcripts. Each transcript was subdivided evenly into 1000 bins and the read coverage was averaged over all the transcripts. **d** Composition of the uniquely mapped fragments, shown as the percentage of fragments in exonic, intronic, and intergenic regions. According to the direction of transcription, exonic and intronic regions were further divided to sense and antisense. **e** Saturation analysis showing the percentage of coding genes recovered at increasing sequencing depth. **f** Saturation analysis showing the percentage of lncRNAs recovered at increasing sequencing depth. **g** Saturation analysis showing the number of splice junctions recovered at increasing sequencing depth. For the purpose of evaluation, the above analyses also include the libraries prepared with the TruSeq Stranded mRNA protocol using the same biological conditions

Increasing numbers of omics studies are being designed to investigate minor cell subpopulations, rare cell types, and even single cells. Effectively executing low-input RNA-Seq is essential to achieve these goals. To determine the applicability of the TaKaRa SMARTer Ultra Low RNA Kit v3 with low-level RNA input--100 or 1000 mESCs from each of three *Zbtb24* knockout (1lox/1lox) clones (biological replicates) and three wild-type (2lox/+) clones (biological replicates), we evaluated its performance by comparing it to that of the TruSeq Stranded mRNA protocol using 2 μg of total RNA, as a “gold standard” that represents overall robustness with regard to rRNA contamination, mRNA species representation, identification of DEGs, and overall reproducibility. The SMARTer kit protocol resulted in libraries with higher levels of rRNA contamination at both the 100 (~ 1 ng RNA) and 1000 cell (~ 10 ng RNA) levels than did the TruSeq Stranded mRNA protocol using standard input RNA amounts (Fig. 6a). The percentage of fragments with both ends mapped to the genome was 91–92% for the TruSeq Stranded mRNA protocol and 60–65% for the SMARTer protocol using either 100 or 1000 cells (Fig. 6b). The coverage of fragments over transcripts suggested the SMARTer protocol libraries were biased toward the 3′-end of transcripts compared to the TruSeq Stranded mRNA protocol libraries (Fig. 6c). For libraries from the SMARTer protocol with 100 and 1000 cells, around 90% of the fragments were from exonic regions, ~ 6% were from intronic regions, and ~ 4% were from intergenic regions, which was comparable to libraries from the TruSeq Stranded mRNA protocol (Fig. 6d). Since the SMARTer protocol is not strand-specific, half of the fragments were from the sense strand and the other half were from the antisense strand of the genes (Fig. 6d). For coding genes, the saturation curves for libraries from the SMARTer protocol with 100 and 1000 cells were very similar and were slightly less robust than those from the TruSeq Stranded mRNA protocol (Fig. 6e). The SMARTer protocol outperformed the TruSeq Stranded mRNA protocol in recovering more lncRNAs at the same sequencing depth (Fig. 6f). However, at the same sequencing depth, the number of splice junctions detected in libraries from the SMARTer protocol was lower than in libraries from the TruSeq Stranded mRNA protocol (Fig. 6g). Overall, low-input RNA samples subjected to the SMARTer protocol, when compared to the TruSeq Stranded mRNA protocol, produced data with greater rRNA contamination but similar rates of exon detection. Furthermore, we recovered fewer coding genes and splice junctions but more lncRNAs from libraries generated with the SMARTer Ultra Low RNA Kit. Overall, the kit performed well on these low-input samples, but as anticipated, did not capture the range of expression recovered with a kit using more input RNA.

### Concordance of expression quantification and DE detection (ultra-low protocol)

**Fig. 7:**
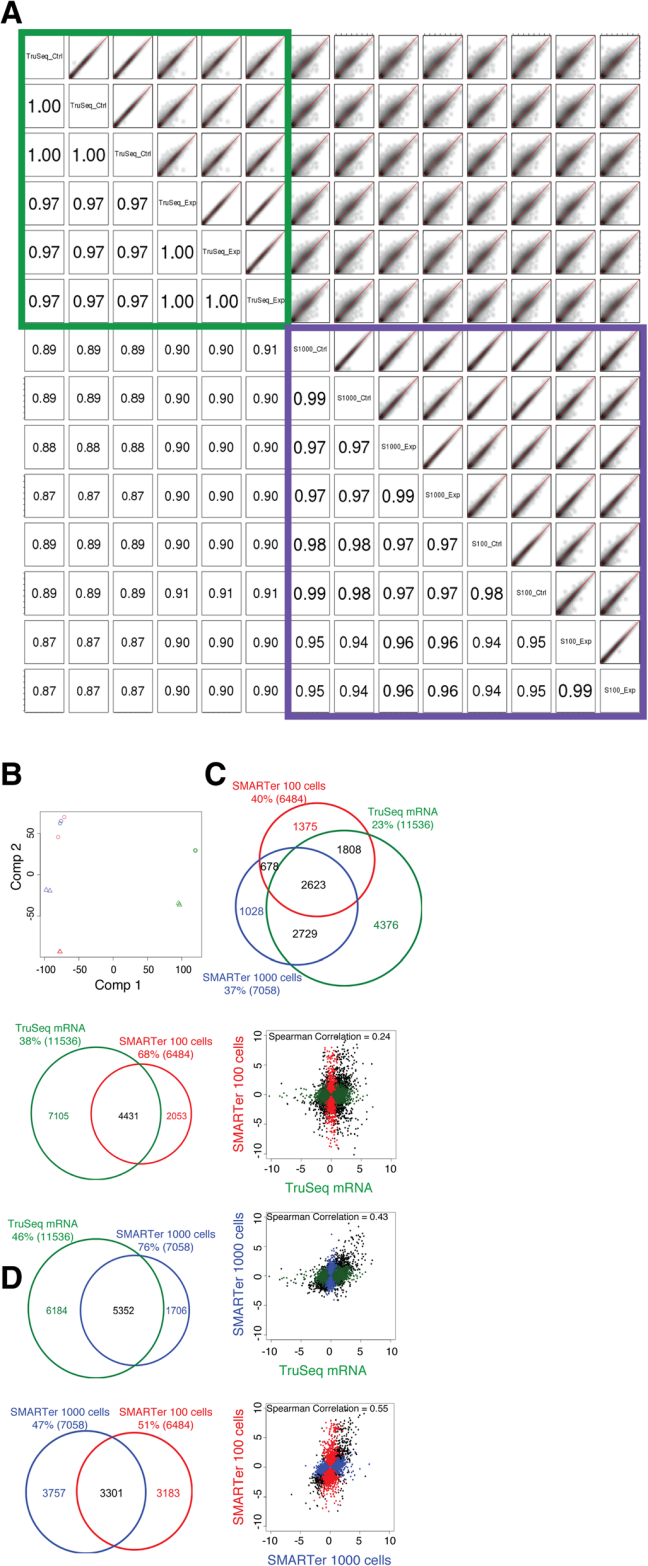
Concordance of expression quantification and DEG detection using the SMARTer Ultra Low RNA Kit. For the purpose of evaluation, the libraries prepared from the same biological conditions with the TruSeq Stranded mRNA protocol are also included. **a** Smoothed color density representation scatter plots (top, right) and Spearman’s rank correlation coefficients (bottom left) for all library pairs using log_2_(cpm + 1) values. 100 and 1000 represent the SMARTer Ultra Low RNA Kit using 100 and 1000 cells. **b** Principal component analysis (PCA) of all libraries using log_2_(cpm + 1) values. Red, blue, and green represent libraries prepared with the ultra-low protocol 100 cells, ultra-low protocol 1000 cells, and TruSeq Stranded mRNA protocol, respectively. Circles and triangles represent control and experimental libraries, respectively. **c** Venn diagram showing the number of DEGs recovered with the SMARTer Ultra Low RNA (100 cells and 1000 cells) and the TruSeq Stranded mRNA kits. **d** Pairwise scatter plots of log_2_ ratio values between the biological conditions using the DEGs. The black dots represent genes called as differentially expressed in libraries prepared with both kits, and the colored dots represent genes called as differentially expressed in libraries from only one kit. The Spearman’s rank correlation coefficient is shown at the top of each plot. The Venn diagram to the left of each scatter plot shows the number of DEGs called for the data produced using both or only one of the protocols

Spearman’s rank correlation coefficients between the low-input samples prepared from the same or different input quantities were very good (0.94–0.99), indicating high reproducibility with the SMARTer Ultra Low RNA Kit protocol. However, the coefficients between samples prepared using the SMARTer and standard TruSeq Stranded mRNA protocols were lower (0.87–0.91) (Fig. 7a). PCA showed that the variability among samples was largely due to differences between the SMARTer and TruSeq Stranded mRNA libraries, as described in the first component (Fig. 7b). The transcriptome profile changes from biological conditions within each protocol could be explained by the second component (Fig. 7b). Further investigation showed the SMARTer protocol tended to allow recovery of genes with higher expression, lower GC content, and shorter length, compared to the TruSeq mRNA protocol (Additional file 7: Figure S7D-F). There were 2623 DEGs shared between the SMARTer libraries generated from either 100 or 1000 cells and the TruSeq Stranded mRNA libraries, accounting for 40, 37, and 23% of the total DEGs detected in each, respectively, but the majority of DEGs recovered from the TruSeq Stranded mRNA libraries (4376 genes) were excluded from the SMARTer libraries (Fig. 7c). The pairwise scatter plots of log_2_ ratios between biological interventions using DEGs showed that the concordance of DEG detection between the SMARTer libraries prepared with 100 cells vs. 1000 cells, or between SMARTer vs. TruSeq Stranded mRNA, was much lower than that between the standard protocols at normal input level (Fig. 7d vs. Figure 5c). In summary, the SMARTer Ultra Low RNA Kit is capable of capturing the effect of biological conditions, but is not as robust as the standard input protocol at a normal input level of 2 μg for the TruSeq Stranded mRNA-Seq protocol.

## Discussion

Comparing global gene expression in differing biological contexts is a cornerstone of contemporary biology. As microarray technology is being supplanted by RNA-Seq methods for many applications, it is imperative to determine which library preparation protocols are best suited for specific needs, for example the recovery of coding vs. non-coding RNAs and reliable discernment of DEGs. Here, we have examined three different standard RNA-Seq library preparation protocols, and one low-input protocol in terms of overall reproducibility, rRNA contamination, read coverage, 5′- and 3′-end bias, and recovery of exonic vs. intronic sequences, lncRNAs, and DEGs. These protocols were the standard input Illumina TruSeq Stranded Total RNA, Illumina TruSeq Stranded mRNA, and modified NuGEN Ovation v2 kits; and the low input TaKaRa SMARTer Low Input RNA-Seq kit v3, tested at two different input levels, 100 (~ 1 ng RNA) and 1000 (~ 10 ng RNA) cells. Although all protocols yielded reproducible data, overall, the Illumina kits generally outperformed the modified NuGEN Ovation v2 kit at standard RNA input levels. The modified NuGEN protocol was useful for the recovery of lncRNAs and intronic sequences, but also had higher levels of rRNA contamination.

### Undesirable recovery of rRNA

One impediment to the efficient recovery of meaningful RNA-Seq data is repetitive rRNA. Nearly 80% of RNA in a cell is rRNA, making it preferable to remove this class of RNA prior to library construction [17]. RNA-Seq library preparation protocols depend on one of two means of reducing rRNA contamination: rRNA depletion and polyA enrichment. For the three standard protocols and the one ultra-low input protocol we evaluated, the TruSeq Stranded Total RNA and the modified NuGEN Ovation RNA-Seq System V2 protocols employ rRNA depletion methods, whereas the TruSeq Stranded mRNA protocol and SMARTer Ultra-low protocol use polyA enrichment methods to reduce rRNA contamination in sequencing libraries. In our present study, the modified NuGEN protocol libraries averaged 15–20% of their reads mapping to rRNA, as compared to 1–5% for the TruSeq protocols (Fig. 2a and Additional file 1: Figure S1A). These results are consistent with those reported by Adiconis et al. (23.2%) [12], but lower than those reported by Shanker et al. (35%) [13]. However, our NuGEN rRNA mapping rates were much higher than those reported by both Sun et al. [18] and Alberti et al. [19] who had only a 1% rRNA mapping rate for both their Illumina- and NuGEN-created libraries. While we cannot explain the differences in rRNA mapping rates for the NuGEN libraries in these studies, in our core facility, the NuGEN Ovation v2 kit libraries consistently resulted in a 15–20% rRNA mapping rate, not only in this study, but also in prior sequencing libraries constructed in our facility (data not shown), thus providing part of the impetus for the current study. We also examined the rRNA mapping rate in libraries prepared from two polyA-enrichment protocols, the Illumina TruSeq Stranded mRNA protocol and the TaKaRa SMARTer Ultra Low RNA protocol. The SMARTer protocol yielded a 7–9% rRNA mapping rate, which was inferior to the TruSeq protocol at standard RNA input levels (1%) (Fig. 6a). The 7–9% mapping rate yielded by the SMARTer protocol in our facility was consistent with that reported by Adiconis et al. [12] and Alberti et al. [19]. Overall, the protocols we tested were able to remove the majority of rRNA. Although the modified NuGen protocol showed relatively higher rRNA content, since the existence of rRNA is not expected to introduce a bias for expression quantification, an increase in sequencing depth would be able to compensate.

### Overall mapping, end bias and exonic coverage

The TruSeq protocols yielded a ≥ 90% overall mapping rate for fragments with both ends mapped to the genome, compared to 60% for the modified NuGEN protocol (Fig. 2b and Additional file 1: Figure S1B). This is on par with a prior study showing NuGEN rRNA-depleted libraries had a 75% alignment rate and TruSeq PolyA-enrichment mRNA libraries had a 90% alignment rate [18].

To assess whether complete transcripts were evenly captured by the three standard library preparation protocols, we examined read coverage over the length of the full transcript. Our results, like those of Acondis [12], indicated that NuGEN libraries displayed augmented 3′-end signal and depleted 5′-end signal, perhaps due to using a combination of both oligo[dT] and random primers during cDNA synthesis [12]. The TruSeq Stranded mRNA libraries were also somewhat biased, as reflected by a lack of reads within 200 bps of the 3′-end, relative to the TruSeq Total RNA libraries (Additional file 2: Figure S2B, 2D). This may be because of the difference between the rRNA depletion approaches used by the TruSeq mRNA and TruSeq total RNA protocols, resulting in more unmappable reads near the 3′-end in TruSeq mRNA libraries due to the presence of polyA tails in these reads.

To determine how well each protocol performed in recovering the transcriptome, we examined the composition of the uniquely mapped fragments from the two Illumina and the modified NuGEN protocols. Ninety percent of our reads were mapped to exons using the TruSeq Stranded mRNA kit, 67–84% using the Total RNA kit, and 35–46% using the NuGEN kit (Fig. 3a and Additional file 3: Figure S3A), which is consistent with similar studies using these kits [9, 11, 13, 18], suggesting that polyA-enrichment protocols may be superior to rRNA depletion protocols for studies focusing on exonic RNA [11, 13, 18]. This is further supported by our finding that, compared to the three standard input protocols, the polyA-based TaKaRa SMARTer Ultra Low RNA Kit had almost the same exonic coverage as the TruSeq Stranded mRNA protocol (Fig. 6d). The inverse was true for the recovery of intronic sequences, with rRNA-depleted libraries outperforming the polyA-enrichment libraries. For example, the modified NuGEN protocol yielded ~ 50% intronic sequences, which was on par with the results of Shanker et al. (after removing PCR duplicates) [13], whereas our TruSeq Stranded Total RNA libraries consisted of 14–28% intronic sequences. In contrast, the TruSeq Stranded mRNA libraries contained only 6–8% intronic sequences (Fig. 3a and Additional file 3: Figure S3A). We also found that the modified NuGEN kit yielded better lncRNA recovery. In this case, better lncRNA recovery may be due to differences in the cDNA synthesis step rather than in the rRNA depletion step: whereas the TruSeq Stranded Total RNA protocol uses only random primers for cDNA synthesis, the modified NuGEN protocol uses a combination of random and oligo [15] primers, thus allowing more efficient capture of both coding and non-coding RNAs with and without polyA-tails [11]. However, it is also possible that some of the lncRNAs identified in the rRNA-depleted libraries are merely false signals originating from intronic reads from other coding genes rather than lncRNAs [11]. Additionally, it is worth noting that in our saturation analysis (Fig. 3b, c Additional file 3: Figure S3B, 3C), the curves reached saturation at ~ 60% coding genes or ~ 30% lncRNAs, suggesting that achieving increased coverage of coding genes or lncRNAs beyond these levels by deeper sequencing would be very difficult.

### Gene quantification and identification of DEGs

Gene expression quantification in and identification of DEGs between samples from different biological conditions are two of the primary goals for most RNA-Seq experiments. In the current study, we identified 960 and 1028 DEGs between experimental and control tumor tissues using the TruSeq Total RNA and mRNA protocols (manuscript in preparation), respectively, which was slightly fewer than the 1430 DEGs identified using the modified NuGEN protocol (Fig. 5b). This contrasts with the work of Sun et al. who recovered fewer DEGs from NuGEN libraries than TruSeq PolyA-enrichement libraries [18]. To explore this difference, we validated our RNA-Seq-identified DEGs using qRT-PCR. We found that a greater proportion of DEGs identified using the TruSeq Stranded Total RNA and mRNA libraries were supported by our qRT-PCR results compared to DEGs identified using the modified NuGEN protocol libraries. That is, the modified NuGEN protocol may have resulted in more false-positive DEGs than did the TruSeq protocols. The comparable performance of the TruSeq Total and mRNA protocols in our study contrasts with the results of Zhao, et al., who directly compared the TruSeq Stranded Total and mRNA protocols using clinical samples. They found the TruSeq Stranded mRNA libraries more accurately predicted gene expression levels than the TruSeq Stranded Total RNA libraries [11].

Although the SMARTer Ultra Low RNA Kit-generated libraries were able to capture the effect of biological differences between experimental and control samples, overall, its performance was inferior to that of the TruSeq Stranded mRNA protocol, given both the higher amount of rRNA recovered and the lower number of DEGs recovered (Figs. 6 and 7). This may be due to the very different levels of input RNA used in these two protocols.

### Limitations and future work

There are still some limitations in this study that could be addressed in future work. For example, this study didn’t include spike-in RNAs, which could serve as a sample independent benchmark to further evaluate the accuracy of DEG detection in libraries prepared by different protocols. Future work could also consider investigating additional ultralow RNA-Seq protocols and using standard RNA samples such as Universal Human Reference RNA (UHRR) for an easier comparison to other studies. [20]

## Conclusions

In summary, all the RNA-Seq library preparation protocols evaluated in this study were suitable for distinguishing between experimental groups when using the manufacturers’ recommended amount of input RNA. However, we made some discoveries that might have been previously overlooked. First, we found that the TruSeq Stranded mRNA protocol is universally applicable to studies focusing on dissecting protein-coding gene profiles when the amount of input RNA is sufficient, whereas the modified NuGEN protocol might provide more information in studies designed to understand lncRNA profiles. Therefore, choosing the appropriate RNA-Seq library preparation protocol for recovering specific classes of RNA should be a part of the overall study design [18]. Second, when dealing with small amounts of input RNA, the SMARTer Ultra Low RNA Kit may be a good choice in terms of rRNA removal, exonic mapping rates and recovered DEGs. Third, our saturation analysis indicated that the required sequencing depth depends on the biological question being addressed by each individual study. Roughly, a minimum of 20 M aligned reads/mate-pairs are required for a project designed to detect coding genes and increasing the sequencing depth to ≥130 M reads may be necessary to thoroughly investigate lncRNAs [21] (note: the needed sequencing depth may also vary depending on different biological samples and study designs). Omics technology and big data will facilitate the development of personalized medicine, but we should understand the outcomes of the experimental parameters and control for those as thoroughly as possible.

## Methods

### Biological samples and RNA isolation

The use of mice in this project has been reviewed and approved by The University of Texas MD Anderson Cancer Center (MD Anderson) IACUC committee (ACUF 04–89-07138, S. Fischer) and (ACUF MODIFICATION 00001124-RN01, T. Chen). C57BL/6 mice were purchased from The Jackson Laboratory (Bar Harbor, ME). For the three standard input RNA-Seq library preparation protocols (Illumina TruSeq Stranded Total RNA, TruSeq Stranded mRNA kit, and the modified NuGEN Ovation RNA-Seq kits), total RNA was isolated from three xenograft tumors (biological replicates) from control [30% calorie restricted diet [19]] and experimental [(diet-induced obese (OB)) xenograft mouse models in the C57BL/6 genetic background, respectively. C57BL/6 mice were chosen, in part, because they are susceptible to obesity when fed a high-fat diet [22]. We fed the mice with two commercial diets following previously established guidelines (Research Diets, Inc., New Brunswick, NJ): a CR diet (D03020702) for lean C57BL/6 mice (30% CR), and a diet-induced obesity (DIO) diet (D12492; consumed ad libitum) for OB C57BL/6 mice, 10 mice per group [23]. Mice were humanely euthanized using carbon dioxide and followed by cervical dislocation, per IACUC approved procedures. A manuscript describing the details of the mouse obesity/tumor xenograft study, including transcriptomic profiling results, is in preparation. For the SMARTer Ultra Low RNA Kit, designed to evaluate both rare cell populations and fixed clinical samples, three mESCs cell lines (biological replicates) from *Zbtb24* knockout (1lox/1lox) clones and three *Zbtb24* wild-type (2lox/+) clones were used as experimental and control samples, respectively. The mice used for this part of the study were generated in-house at MD Anderson Science Park. A manuscript describing the *Zbtb24* KO mESCs, including transcriptomic profiling results, is also in preparation.

Total RNA from mouse xenograft tumor tissues was isolated using TRIZOL following the manufacturer’s protocol. Isolated RNA samples were treated with DNase I followed by purification with a QIAGEN RNeasy Mini kit (Madison, WI). Total RNA from mESCs was extracted using the QIAGEN RNeasy Mini kit with on-column DNase treatment following the manufacturer’s protocol. Both concentration and quality of all the isolated RNA samples were measured and checked with an Agilent Bioanalyzer 2100 and Qubit. All RNA samples had RNA integrity numbers > 8.90. For the low-cell-input experiments, 100 cells and 1000 cells (~ 1 and 10 ng RNA, respectively, according to the SMARTer Ultra Low RNA kit user manual) were used directly without isolating total RNA in accordance with manufacturer recommendations.

### TruSeq stranded total RNA and mRNA library preparations

Libraries were prepared using the Illumina TruSeq Stranded Total RNA (Cat. # RS-122-2301) or mRNA (Cat. # RS-122-2101) kit according to the manufacturer’s protocol starting with 1 μg total RNA. Briefly, rRNA-depleted RNAs (Total RNA kit) or purified mRNAs (mRNA kit) were fragmented and converted to cDNA with reverse transcriptase. The resulting cDNAs were converted to double stranded cDNAs and subjected to end-repair, A-tailing, and adapter ligation. The constructed libraries were amplified using 8 cycles of PCR.

### NuGEN ovation RNA-Seq system v2 modified with SPRI-TE library construction system

Total RNA (100 ng) was converted to cDNA using the NuGEN Ovation RNA-Seq System v2 (Cat. # 7102–32) (NuGEN) following the manufacturer’s protocol (NuGEN, San Carlos, CA). NuGEN-amplified double-stranded cDNAs were broken into ~ 180 base pair (bp) fragments by sonication with a Covaris S220 instrument (Covaris, Woburn, MA). Fragmented cDNAs were processed on a SPRI-TE library construction system (Beckman Coulter, Fullerton, CA). Uniquely indexed NEXTflex adapters (Bioo Scientific, Austin, TX) were ligated onto each sample to allow for multiplexing. Adapter-ligated libraries were amplified [1 cycle at 98 °C for 45 s; 15 cycles at 98 °C for 15 s, 65 °C for 30 s, and 72 °C for 30 s; 1 cycle at 72 °C for 1 min; and a hold at 4 °C] using a KAPA library amplification kit (KAPA Biosystems, Wilmington, MA) and purified with AMPure XP beads (Beckman Coulter).

### Modified protocol for the SMARTer ultra low RNA and Nextera DNA library preparation kits

mESC were lysed in the reaction buffer included in the SMARTer Ultra Low RNA Kit v3 (Cat. # 634849) (TaKaRa, Japan). cDNA was then synthesized using the SMARTer Ultra Low RNA Kit followed by library construction using the Nextera DNA Sample Preparation Kit (Cat. # FC-131-1024) (Illumina, San Diego, CA), according to the manufacturers’ protocols. We performed 10 cycles of PCR for 1000 cells (~ 10 ng RNA) (SMARTer 1000), and 18 cycles of PCR for 100 cells (~ 1 ng RNA) (SMARTer 100).

### Next-generation sequencing

Ten pM of pooled libraries were processed using a cBot (Illumina) for cluster generation before sequencing on an Illumina HiSeq 2500 (2 × 76 bp run).

### RNA-Seq data analysis

#### Mapping

Reads were mapped to rRNA sequences (GI numbers: 262231778, 120444901, 120444900, 328447215, 38176281 and Ensembl IDs: ENSMUST00000082388, ENSMUST00000082390, ENSMUST00000083988, ENSMUST00000157970) using Bowtie2 (version 2.1.0) [24]. Reads that were not mapped to rRNAs were then mapped to the mouse genome (mm10) using TopHat (version 2.0.10) [25].

#### Read coverage over transcripts

The longest transcript from each gene was chosen to represent the gene. The reads were then mapped to all the transcript sequences using Bowtie2. Transcripts with fewer than 200 total fragment counts or shorter than 1000 bps were filtered out leaving at least 12 k transcripts for each sample. Each full-length transcript was subdivided evenly into 1000 bins. The mean coverage of fragments over each bin was normalized to the total coverage over the whole transcript and then averaged over all the transcripts. Alternatively, the coverage of fragments over each position of the 1000 bps downstream of the 5′-end or upstream of the 3′-end was normalized by the mean coverage of the whole transcript, and then averaged over all the transcripts.

#### Discovery of splicing junctions

The number of known splicing junctions (defined as junctions with both 5′- and 3′- splice sites annotated in the reference gene set) supported by at least one read in each sample was counted using RSeQC (version 2.6.4) [26].

#### Saturation plots

Each point in a saturation curve was generated by randomly selecting the desired number of fragments and calculating the percentage of genes with more than 10 fragments over all the genes. For each sample, this procedure was repeated three times and the curve represents the average percentage of genes at each corresponding number of fragments.

#### Sample clustering

Hierarchical clustering of samples was performed using the log_2_(cpm + 1) values of all the genes using the dist function and Euclidean method in R, as well as the hierarchical clustering (hclust) function and complete method in R.

#### Differential expression

The number of fragments in each known gene from GENCODE Release M4 [27] was enumerated using the htseq-count script within the HTSeq package (version 0.6.1) [28] with options -m union and -s no/reverse (“no” for strand-unspecific protocols and “yes” for strand-specific protocols). Fragments that were mapped to multiple genes or multiple locations were discarded. For strand-specific protocols, fragments that were mapped to the antisense strand of the genes were discarded. Genes represented by fewer than 10 fragments in all samples were removed before performing differential expression analysis. Differences in gene expression between conditions were statistically assessed using the R/Bioconductor package edgeR (version 3.6.1) [29]. Genes with a false discovery rate (FDR) ≤ 0.05 and length > 200 bps were called as differentially expressed. The software used in this study is listed in Table 1.

**Table 1:** Software used in this study

software	version	website	reference
Bowtie2	2.1.0	http://bowtie-bio.sourceforge.net/bowtie2/index.shtml	Fast gapped-read alignment with Bowtie 2
TopHat	2.0.10	http://ccb.jhu.edu/software/tophat/index.shtml	TopHat: discovering splice junctions with RNA-Seq
HTSeq-count	0.6.1	https://htseq.readthedocs.io	HTSeq — A Python framework to work with high-throughput sequencing data
edgeR	3.6.1	https://bioconductor.org/packages/release/bioc/html/edgeR.html	edgeR: a Bioconductor package for differential expression analysis of digital gene expression data
RSeQC	2.6.4	http://rseqc.sourceforge.net	RSeQC: quality control of RNA-seq experiments
R	3.1.0	https://www.r-project.org	

#### Box plots of gene expression, GC content and gene length

Between a pair of protocols, the genes with elevated expression in one protocol compared to the other protocol were identified by edgeR at FDR < 0.01 and log_2_ ratio > 1. Then the gene expression, GC content, and gene length for the two groups of more highly expressed genes were plotted in box plots. The gene expression is the average FPKM (number of fragments per kilobase per million mapped fragments) value of all the samples used in the evaluation of the standard input or ultralow input protocols. The longest transcript representing each gene was used to calculate both gene GC content and length.

## Additional files

Additional file 1: **Figure S1.** Mapping statistics and read coverage over transcripts for all the libraries prepared from 100 ng RNA with standard input protocols prepared. A. The rRNA mapping rate was calculated as the percentage of fragments that were mappable to rRNA sequences. B. The non-rRNA mapping rate was calculated from all the non-rRNA fragments as the percentage of fragments with both ends or one end mapped to the genome. C. Multiple alignment rates were determined from non-rRNA fragments that were mapped to multiple locations of the genome. D. Read-bias was assessed using the read coverage over transcripts. Each transcript was subdivided evenly into 1000 bins and the read coverage was averaged over all the transcripts. (TIF 2856 kb)

Additional file 2: **Figure S2.** Read coverage near the 5′- (A and C) and 3′-end (B and D) of the transcripts. The TruSeq Total RNA and mRNA libraries shown in A and B were prepared from 1 μg RNA and in C and D were prepared from 100 ng RNA. The read coverage over each position of the 1000 bps downstream of the 5′-end or upstream of the 3′-end was normalized to the mean coverage over the whole transcript, and then averaged over all the transcripts. (TIF 2833 kb)

Additional file 3: **Figure S3.** Representation of the transcriptome for all the libraries prepared from 100 ng RNA with standard input protocols. A. Composition of the uniquely mapped fragments, shown as the percentage of fragments in exonic, intronic, and intergenic regions. According to the direction of transcription, exonic and intronic regions were further divided into sense and antisense. B. Saturation analysis showing the percentage of coding genes recovered (calculated as the genes with more than 10 fragments) at increasing sequencing depth. C-D. Saturation analysis showing the percentage of lncRNAs recovered (calculated as the lncRNAs with more than 10 fragments) at increasing sequencing depth. In C, the six libraries created using each of three protocols (18 libraries total) are plotted individually. In D, the six libraries from the same protocol were pooled. E. Saturation analysis showing the number of splice junctions recovered at increasing sequencing depth. (TIF 3724 kb)

Additional file 4: **Figure S4.** Concordance of expression quantification between the libraries prepared from 100 ng RNA with standard input protocols. A. Scatter plots in a smoothed color density representation (top-right panel) and Spearman’s rank correlation coefficients (bottom-left panel) for all pairs of libraries using log_2_(cpm + 1) values. B. Unsupervised clustering of all the libraries using log_2_(cpm + 1) values. Euclidean distance with complete linkage was used to cluster the libraries. C. Principal component analysis (PCA) of all the libraries, using log_2_(cpm + 1) values. The values for each gene across all the libraries were centered to zero and scaled to have unit variance before being analyzed. Circles and triangles represent control and experimental libraries, respectively (NuGEN, red; TruSeq mRNA, green; TrueSeq Total RNA, blue). For all analyses in Fig. 4, genes represented by fewer than 10 fragments in all the libraries were excluded. (TIF 7558 kb)

Additional file 5: **Figre S5.** Concordance of expression quantification using standard protocols with additional technical replicates prepared by the TruSeq Stranded Total RNA and mRNA protocols. A. Unsupervised clustering of all the libraries using log_2_(cpm + 1) values. Euclidean distance with complete linkage was used to cluster the libraries. B. Principal component analysis (PCA) for all libraries using log_2_(cpm + 1) values. Blue, green and red dots represent libraries prepared using the TruSeq Stranded Total RNA, TruSeq Stranded mRNA, and NuGen protocols, respectively. The darker colors represent the original libraries presented in this study, and the lighter colors are technical replicates prepared at different times. Circles and triangles represent control and experimental libraries, respectively. (TIF 2607 kb)

Additional file 6: **Figure S6.** Supplementary to Fig. 5. A. Principle component analysis (PCA) for the libraries prepared with the TruSeq Total RNA (100 ng) and the TruSeq mRNA (100 ng) protocols. B. (Left) Venn diagram showing the number of DEGs recovered using the specified protocols. The modified NuGEN protocol is not included for the comparison, because one of the libraries prepared with the TruSeq Total RNA protocol (100 ng) and the TruSeq mRNA protocol (100 ng) used a different xenograft tumor from a different mouse. [9] Pairwise scatter plots of log_2_ ratios between tumor tissues of control and experimental mice based on DEGs. The black dots represent genes that were called as differentially expressed regardless of library preparation method, and colored dots represent genes that were called as differentially expressed with only one library preparation method. The Spearman’s rank correlation coefficient is shown at the top of the plot. (TIF 1431 kb)

Additional file 7: **Figure S7.** Box plots of gene expression, GC content and gene length for the genes with elevated expression estimation in one protocol compared to the other protocol. Top figures are box plots of gene expression in log_2_(FPKM+ 1). Middle figures are box plots of GC content. Bottom figures are box plots of gene length. Panels A-C are for the standard input methods. Panels D-F are for the SMARTer Ultra Low RNA Kit. Panel A shows TruSeq mRNA protocol vs. the TruSeq Total RNA protocol. Panel B shows the TruSeq mRNA protocol vs. the modified NuGEN protocol. Panel C shows the NuGEN protocol vs. the TruSeq Total RNA protocol. Panel D shows the SMARTer Ultra Low RNA Kit 100 cells vs. 1000 cells. Panel E shows the TruSeq mRNA protocol vs. the SMARTer ultra-low protocol (100 cells). Panel F shows the TruSeq mRNA protocol vs. the SMARTer protocol (1000 cells). (TIF 2445 kb)

## Data Availability

The raw dataset for the ultralow protocol has been deposited in GEO and can be accessed by the accession number GSE131398. The other datasets for the standard input protocols are still being analyzed for a manuscript in preparation. They will be deposited and made available at GEO after the manuscript is submitted. Until then, the datasets are available from the corresponding author on reasonable request.

## Abbreviations

ABRF: Association of Biomolecular Resource Facilities
cpm: Count per million fragments mapped to exons
DEGs: Differentially expressed genes
ERCC: External RNA Controls Consortium
FDR: False discovery rate
FPKM: Fragments per kilobase per million
GEO: Gene Expression Omnibus
hclust: Hierarchical clustering
hts: High-throughput sequencing
lncRNAs: Long non-coding RNAs
MD Anderson: The University of Texas MD Anderson Cancer Center
mESCs: Mouse embryonic stem cells
NGS: Next-generation sequencing
PCA: Principal component analysis
qPCR: Quantitative PCR
RNA-Seq: Ribonucleic acid sequencing
rRNA: Ribosomal RNA

## References

[1] Margulies M, Egholm M, Altman WE, Attiya S, Bader JS, Bemben LA (2005). Genome sequencing in microfabricated high-density picolitre reactors. Nature..

[2] Mardis ER (2008). Next-generation DNA sequencing methods. Annu Rev Genomics Hum Genet.

[3] Mortazavi A, Williams BA, McCue K, Schaeffer L, Wold B (2008). Mapping and quantifying mammalian transcriptomes by RNA-Seq. Nat Methods.

[4] Nagalakshmi U, Wang Z, Waern K, Shou C, Raha D, Gerstein M (2008). The transcriptional landscape of the yeast genome defined by RNA sequencing. Science..

[5] Lister R, O'Malley RC, Tonti-Filippini J, Gregory BD, Berry CC, Millar AH (2008). Highly integrated single-base resolution maps of the epigenome in Arabidopsis. Cell..

[6] Barrett Tanya, Wilhite Stephen E., Ledoux Pierre, Evangelista Carlos, Kim Irene F., Tomashevsky Maxim, Marshall Kimberly A., Phillippy Katherine H., Sherman Patti M., Holko Michelle, Yefanov Andrey, Lee Hyeseung, Zhang Naigong, Robertson Cynthia L., Serova Nadezhda, Davis Sean, Soboleva Alexandra (2012). NCBI GEO: archive for functional genomics data sets—update. Nucleic Acids Research.

[7] Oliver HF, Orsi RH, Ponnala L, Keich U, Wang W, Sun Q (2009). Deep RNA sequencing of L. monocytogenes reveals overlapping and extensive stationary phase and sigma B-dependent transcriptomes, including multiple highly transcribed noncoding RNAs. BMC Genomics.

[8] Consortium SM-I (2014). A comprehensive assessment of RNA-seq accuracy, reproducibility and information content by the sequencing quality control consortium. Nat Biotechnol.

[9] Li S, Tighe SW, Nicolet CM, Grove D, Levy S, Farmerie W (2014). Multi-platform assessment of transcriptome profiling using RNA-seq in the ABRF next-generation sequencing study. Nat Biotechnol.

[10] Schuierer S, Carbone W, Knehr J, Petitjean V, Fernandez A, Sultan M (2017). A comprehensive assessment of RNA-seq protocols for degraded and low-quantity samples. BMC Genomics.

[11] Zhao S, Zhang Y, Gamini R, Zhang B, von Schack D (2018). Evaluation of two main RNA-seq approaches for gene quantification in clinical RNA sequencing: polyA+ selection versus rRNA depletion. Sci Rep.

[12] Adiconis X, Borges-Rivera D, Satija R, DeLuca DS, Busby MA, Berlin AM (2013). Comparative analysis of RNA sequencing methods for degraded or low-input samples. Nat Methods.

[13] Shanker S, Paulson A, Edenberg HJ, Peak A, Perera A, Alekseyev YO (2015). Evaluation of commercially available RNA amplification kits for RNA sequencing using very low input amounts of total RNA. J Biomol Tech.

[14] Wu AR, Neff NF, Kalisky T, Dalerba P, Treutlein B, Rothenberg ME (2014). Quantitative assessment of single-cell RNA-sequencing methods. Nat Methods.

[15] Ziegenhain C, Vieth B, Parekh S, Reinius B, Guillaumet-Adkins A, Smets M (2017). Comparative analysis of single-cell RNA sequencing methods. Mol Cell.

[16] Roy B, Haupt LM, Griffiths LR (2013). Review: alternative splicing (AS) of genes as an approach for generating protein complexity. Current genomics.

[17] O'Neil D, Glowatz H, Schlumpberger M. Ribosomal RNA depletion for efficient use of RNA-seq capacity. Current protocols in molecular biology. 2013;Chapter 4:Unit 4 19. 10.1002/0471142727.mb0419s103. PubMed PMID: 23821444. 10.1002/0471142727.mb0419s103 23821444

[18] Sun Z, Asmann YW, Nair A, Zhang Y, Wang L, Kalari KR (2013). Impact of library preparation on downstream analysis and interpretation of RNA-Seq data: comparison between Illumina PolyA and NuGEN ovation protocol. PLoS One.

[19] Alberti A, Belser C, Engelen S, Bertrand L, Orvain C, Brinas L (2014). Comparison of library preparation methods reveals their impact on interpretation of metatranscriptomic data. BMC Genomics.

[20] Munro SA, Lund SP, Pine PS, Binder H, Clevert DA, Conesa A (2014). Assessing technical performance in differential gene expression experiments with external spike-in RNA control ratio mixtures. Nat Commun.

[21] Sims D, Sudbery I, Ilott NE, Heger A, Ponting CP (2014). Sequencing depth and coverage: key considerations in genomic analyses. Nat Rev Genet.

[22] Corbett TH, Roberts BJ, Leopold WR, Peckham JC, Wilkoff LJ, Griswold DP (1984). Induction and chemotherapeutic response of two transplantable ductal adenocarcinomas of the pancreas in C57BL/6 mice. Cancer Res.

[23] Lashinger Laura M., Malone Lauren M., McArthur Mark J., Goldberg Jason A., Daniels Elizabeth A., Pavone Amy, Colby Jennifer K., Smith Nicole C., Perkins Susan N., Fischer Susan M., Hursting Stephen D. (2011). Genetic Reduction of Insulin-like Growth Factor-1 Mimics the Anticancer Effects of Calorie Restriction on Cyclooxygenase-2–Driven Pancreatic Neoplasia. Cancer Prevention Research.

[24] Langmead B, Salzberg SL (2012). Fast gapped-read alignment with bowtie 2. Nat Methods.

[25] Trapnell C, Pachter L, Salzberg SL (2009). TopHat: discovering splice junctions with RNA-Seq. Bioinformatics..

[26] Wang L, Wang S, Li W (2012). RSeQC: quality control of RNA-seq experiments. Bioinformatics..

[27] Harrow J, Denoeud F, Frankish A, Reymond A, Chen CK, Chrast J (2006). GENCODE: producing a reference annotation for ENCODE. Genome Biol.

[28] Anders S, Pyl PT, Huber W (2015). HTSeq--a Python framework to work with high-throughput sequencing data. Bioinformatics..

[29] Robinson MD, McCarthy DJ, Smyth GK (2010). edgeR: a Bioconductor package for differential expression analysis of digital gene expression data. Bioinformatics..

