# Supplementary figures and images for: Systematic evaluation of RNA-Seq preparation protocol performance
Source: BMC Genomics. 2019 Jul 11;20:571. doi: 10.1186/s12864-019-5953-1 (PMC6625085; doi:10.1186/s12864-019-5953-1)

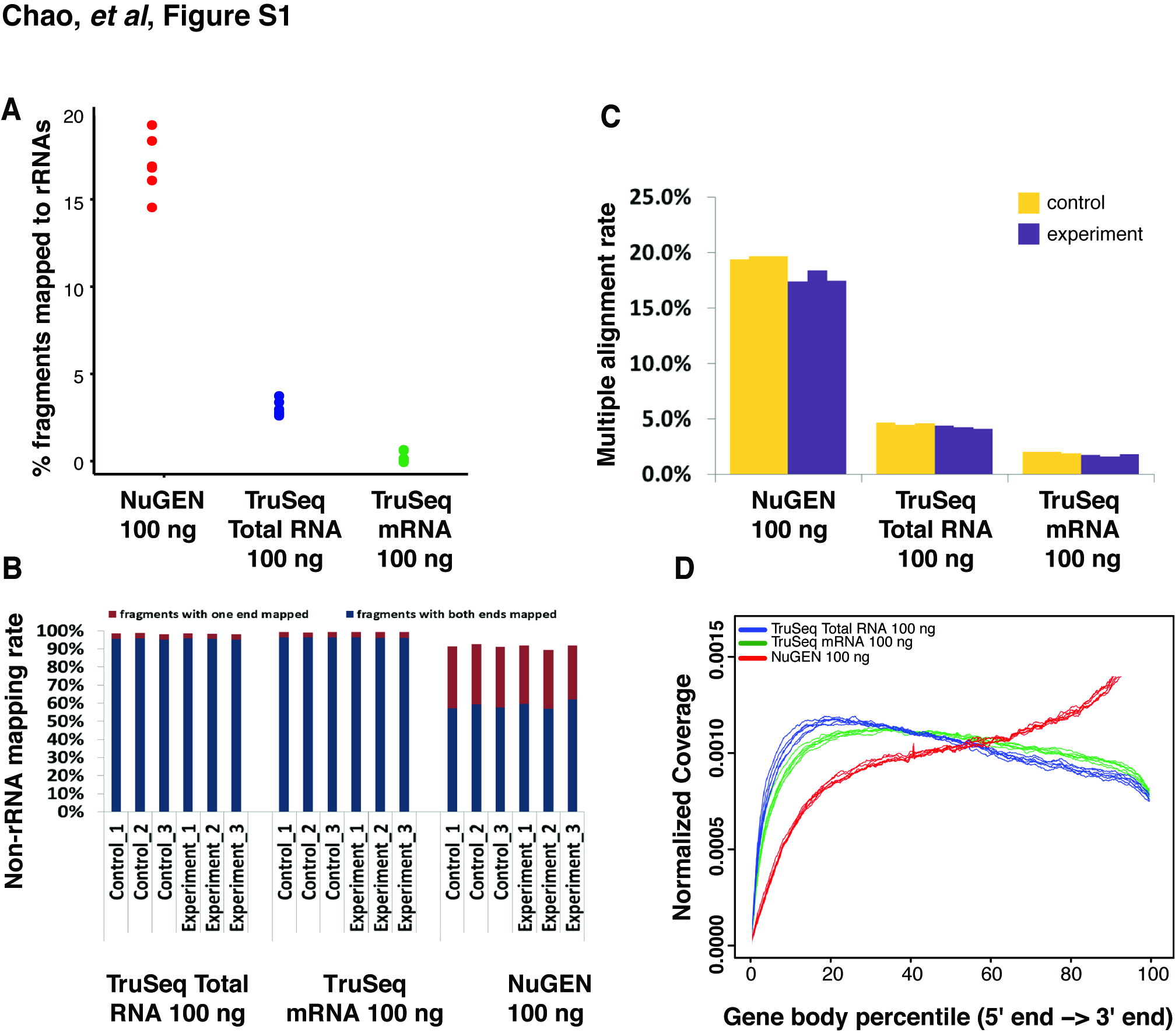

Supplement: Supplementary file 1 — Figure S1. Mapping statistics and read coverage over transcripts for all the libraries prepared from 100 ng RNA with standard input protocols prepared. A. The rRNA mapping rate was calculated as the percentage of fragments that were mappable to rRNA sequences. B. The non-rRNA mapping rate was calculated from all the non-rRNA fragments as the percentage of fragments with both ends or one end mapped to the genome. C. Multiple alignment rates were determined from non-rRNA fragments that were mapped to multiple locations of the genome. D. Read-bias was assessed using the read coverage over transcripts. Each transcript was subdivided evenly into 1000 bins and the read coverage was averaged over all the transcripts. (TIF 2856 kb) [file 12864_2019_5953_MOESM1_ESM.tif]

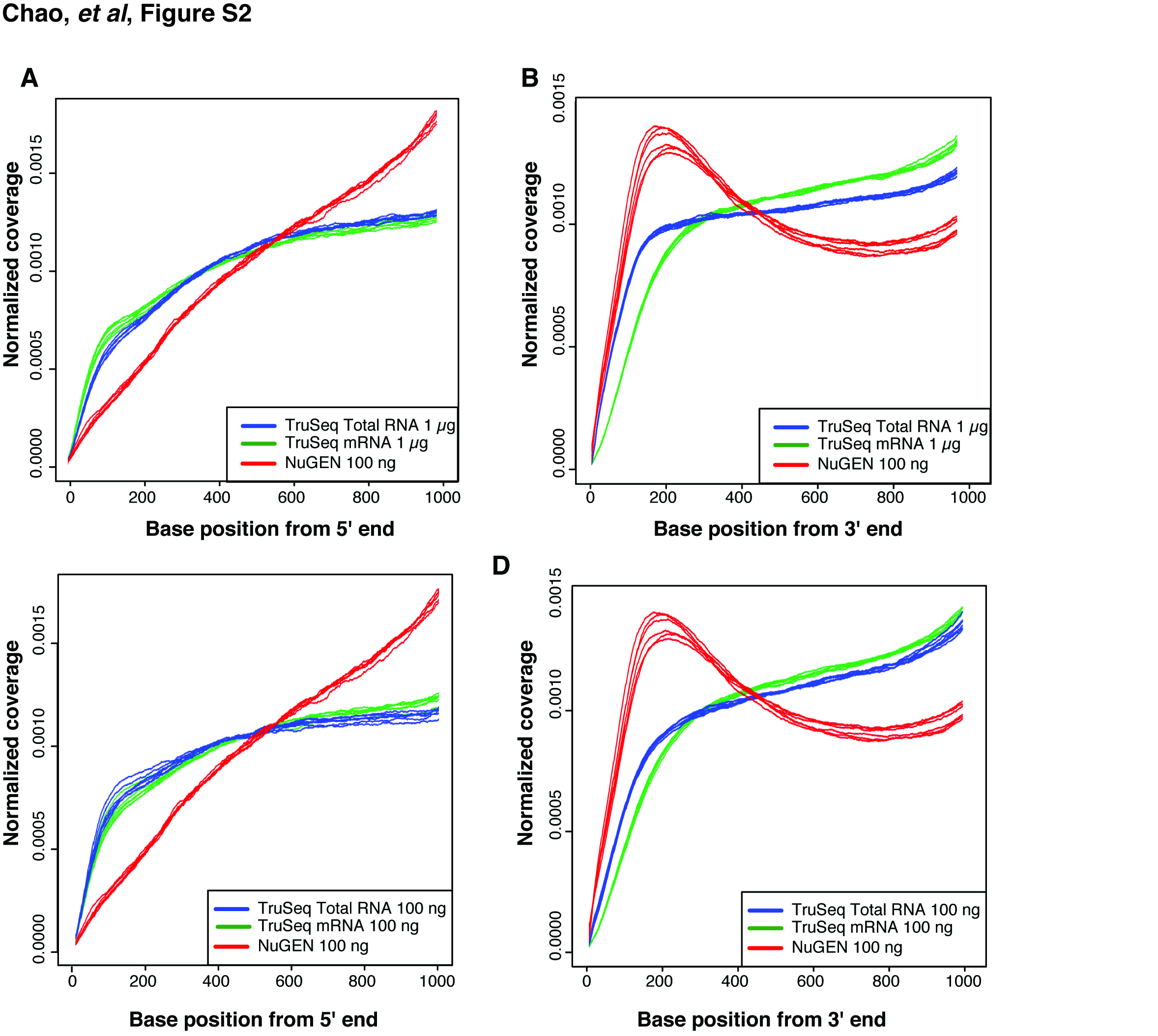

Supplement: Supplementary file 2 — Figure S2. Read coverage near the 5′- (A and C) and 3′-end (B and D) of the transcripts. The TruSeq Total RNA and mRNA libraries shown in A and B were prepared from 1 μg RNA and in C and D were prepared from 100 ng RNA. The read coverage over each position of the 1000 bps downstream of the 5′-end or upstream of the 3′-end was normalized to the mean coverage over the whole transcript, and then averaged over all the transcripts. (TIF 2833 kb) [file 12864_2019_5953_MOESM2_ESM.tif]

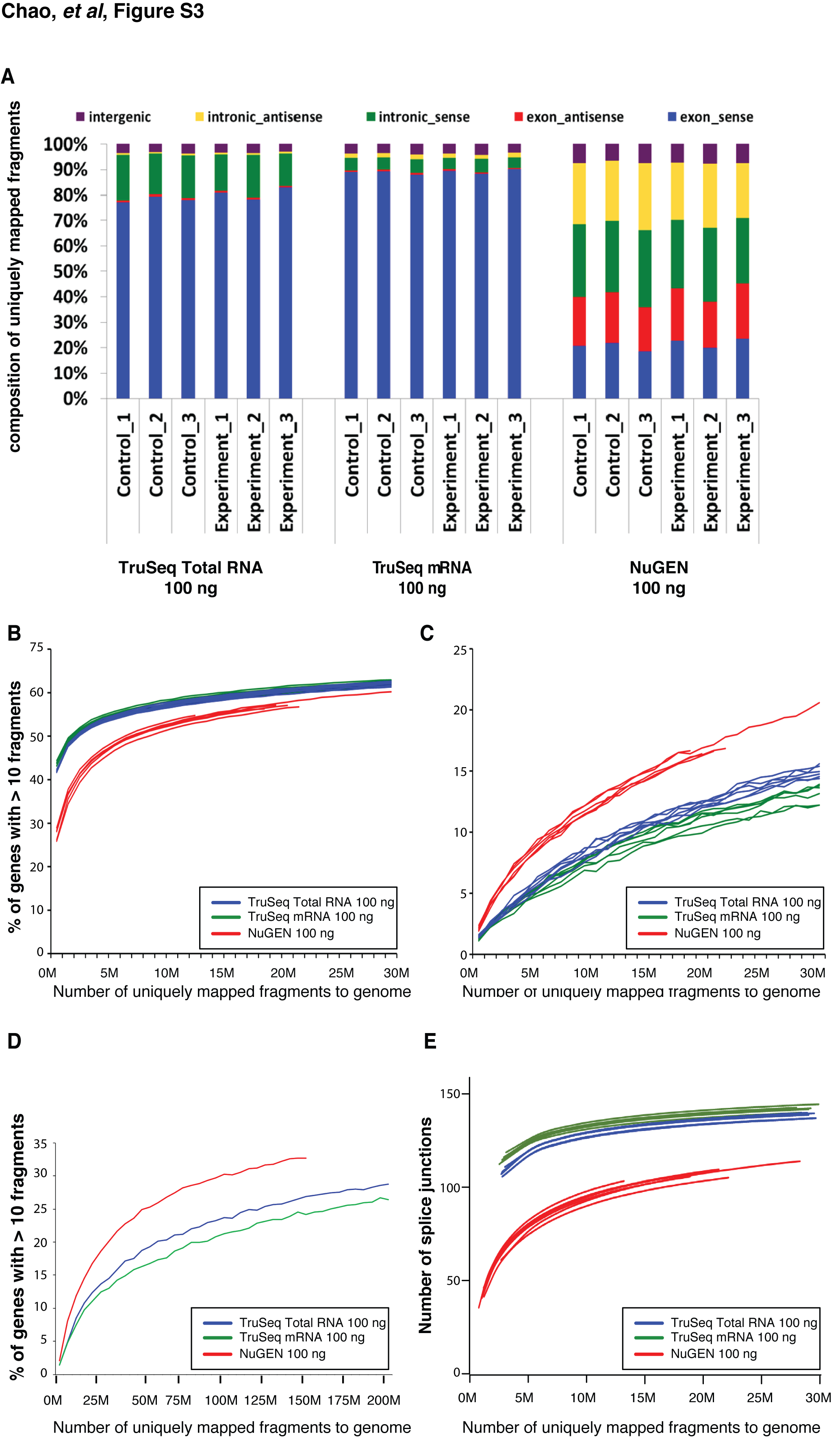

Supplement: Supplementary file 3 — Figure S3. Representation of the transcriptome for all the libraries prepared from 100 ng RNA with standard input protocols. A. Composition of the uniquely mapped fragments, shown as the percentage of fragments in exonic, intronic, and intergenic regions. According to the direction of transcription, exonic and intronic regions were further divided into sense and antisense. B. Saturation analysis showing the percentage of coding genes recovered (calculated as the genes with more than 10 fragments) at increasing sequencing depth. C-D. Saturation analysis showing the percentage of lncRNAs recovered (calculated as the lncRNAs with more than 10 fragments) at increasing sequencing depth. In C, the six libraries created using each of three protocols (18 libraries total) are plotted individually. In D, the six libraries from the same protocol were pooled. E. Saturation analysis showing the number of splice junctions recovered at increasing sequencing depth. (TIF 3724 kb) [file 12864_2019_5953_MOESM3_ESM.tif]

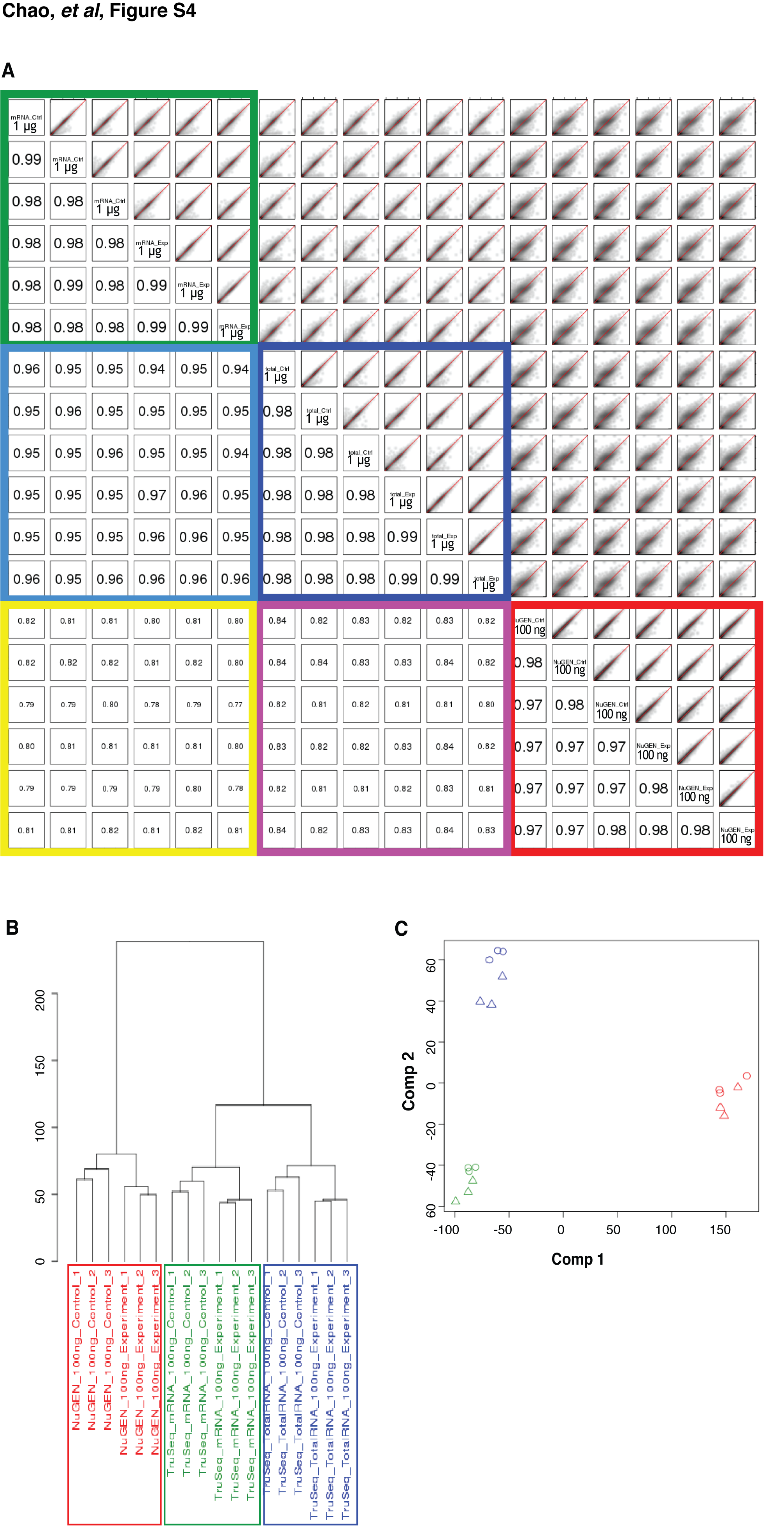

Supplement: Supplementary file 4 — Figure S4. Concordance of expression quantification between the libraries prepared from 100 ng RNA with standard input protocols. A. Scatter plots in a smoothed color density representation (top-right panel) and Spearman’s rank correlation coefficients (bottom-left panel) for all pairs of libraries using log2(cpm + 1) values. B. Unsupervised clustering of all the libraries using log2(cpm + 1) values. Euclidean distance with complete linkage was used to cluster the libraries. C. Principal component analysis (PCA) of all the libraries, using log2(cpm + 1) values. The values for each gene across all the libraries were centered to zero and scaled to have unit variance before being analyzed. Circles and triangles represent control and experimental libraries, respectively (NuGEN, red; TruSeq mRNA, green; TrueSeq Total RNA, blue). For all analyses in Fig. 4, genes represented by fewer than 10 fragments in all the libraries were excluded. (TIF 7558 kb) [file 12864_2019_5953_MOESM4_ESM.tif]

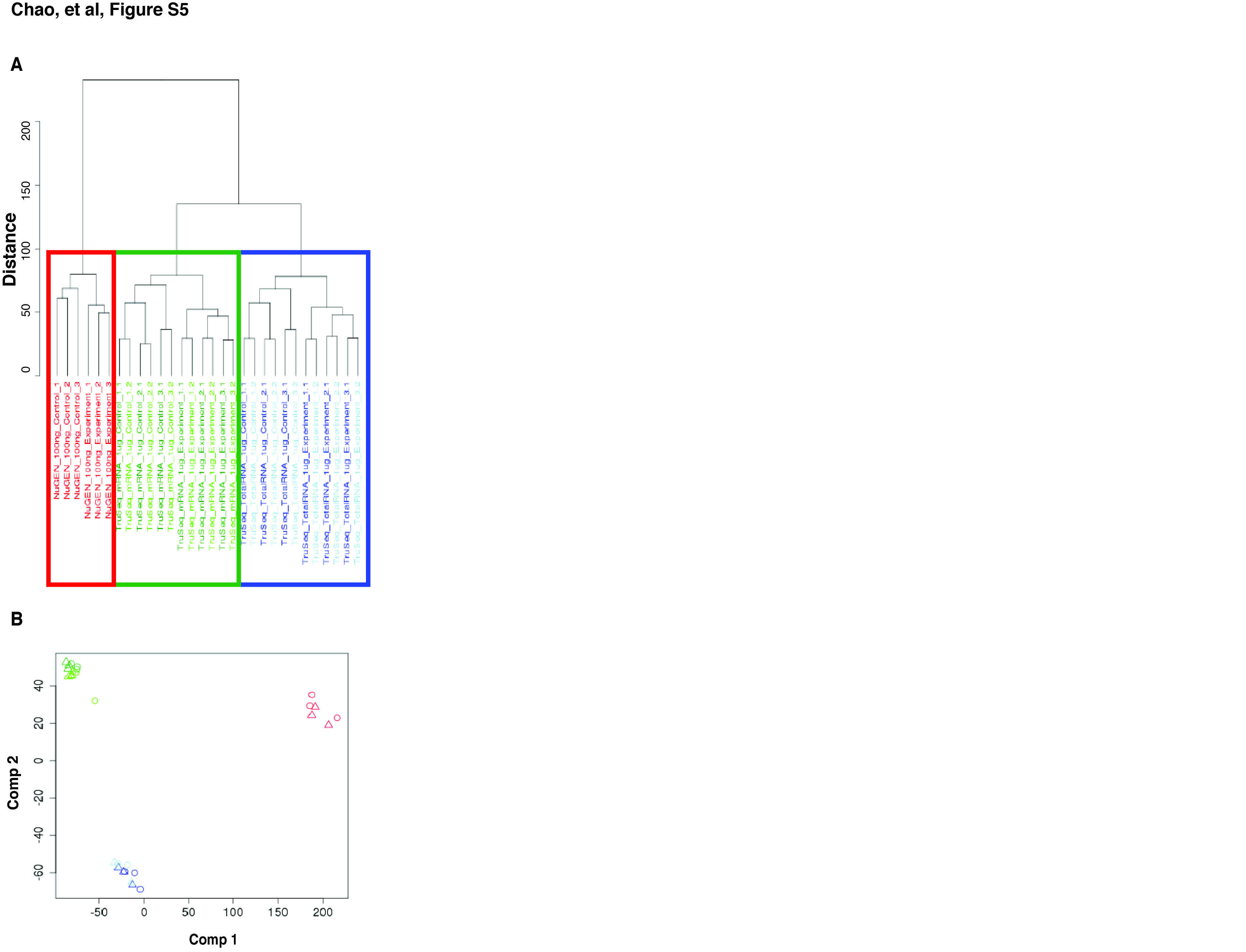

Supplement: Supplementary file 5 — Figre S5. Concordance of expression quantification using standard protocols with additional technical replicates prepared by the TruSeq Stranded Total RNA and mRNA protocols. A. Unsupervised clustering of all the libraries using log2(cpm + 1) values. Euclidean distance with complete linkage was used to cluster the libraries. B. Principal component analysis (PCA) for all libraries using log2(cpm + 1) values. Blue, green and red dots represent libraries prepared using the TruSeq Stranded Total RNA, TruSeq Stranded mRNA, and NuGen protocols, respectively. The darker colors represent the original libraries presented in this study, and the lighter colors are technical replicates prepared at different times. Circles and triangles represent control and experimental libraries, respectively. (TIF 2607 kb) [file 12864_2019_5953_MOESM5_ESM.tif]

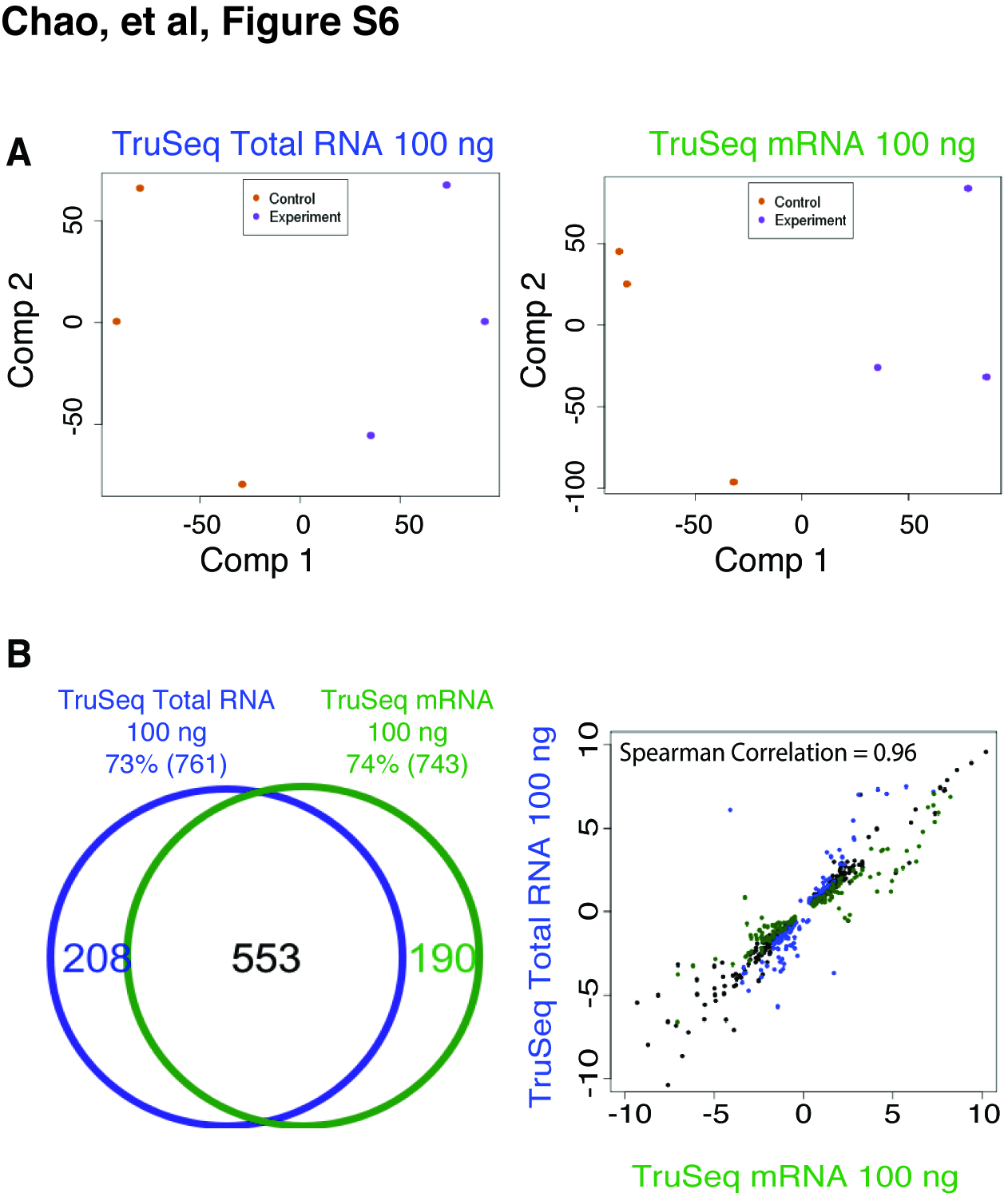

Supplement: Supplementary file 6 — Figure S6. Supplementary to Fig. 5. A. Principle component analysis (PCA) for the libraries prepared with the TruSeq Total RNA (100 ng) and the TruSeq mRNA (100 ng) protocols. B. (Left) Venn diagram showing the number of DEGs recovered using the specified protocols. The modified NuGEN protocol is not included for the comparison, because one of the libraries prepared with the TruSeq Total RNA protocol (100 ng) and the TruSeq mRNA protocol (100 ng) used a different xenograft tumor from a different mouse. [9] Pairwise scatter plots of log2 ratios between tumor tissues of control and experimental mice based on DEGs. The black dots represent genes that were called as differentially expressed regardless of library preparation method, and colored dots represent genes that were called as differentially expressed with only one library preparation method. The Spearman’s rank correlation coefficient is shown at the top of the plot. (TIF 1431 kb) [file 12864_2019_5953_MOESM6_ESM.tif]

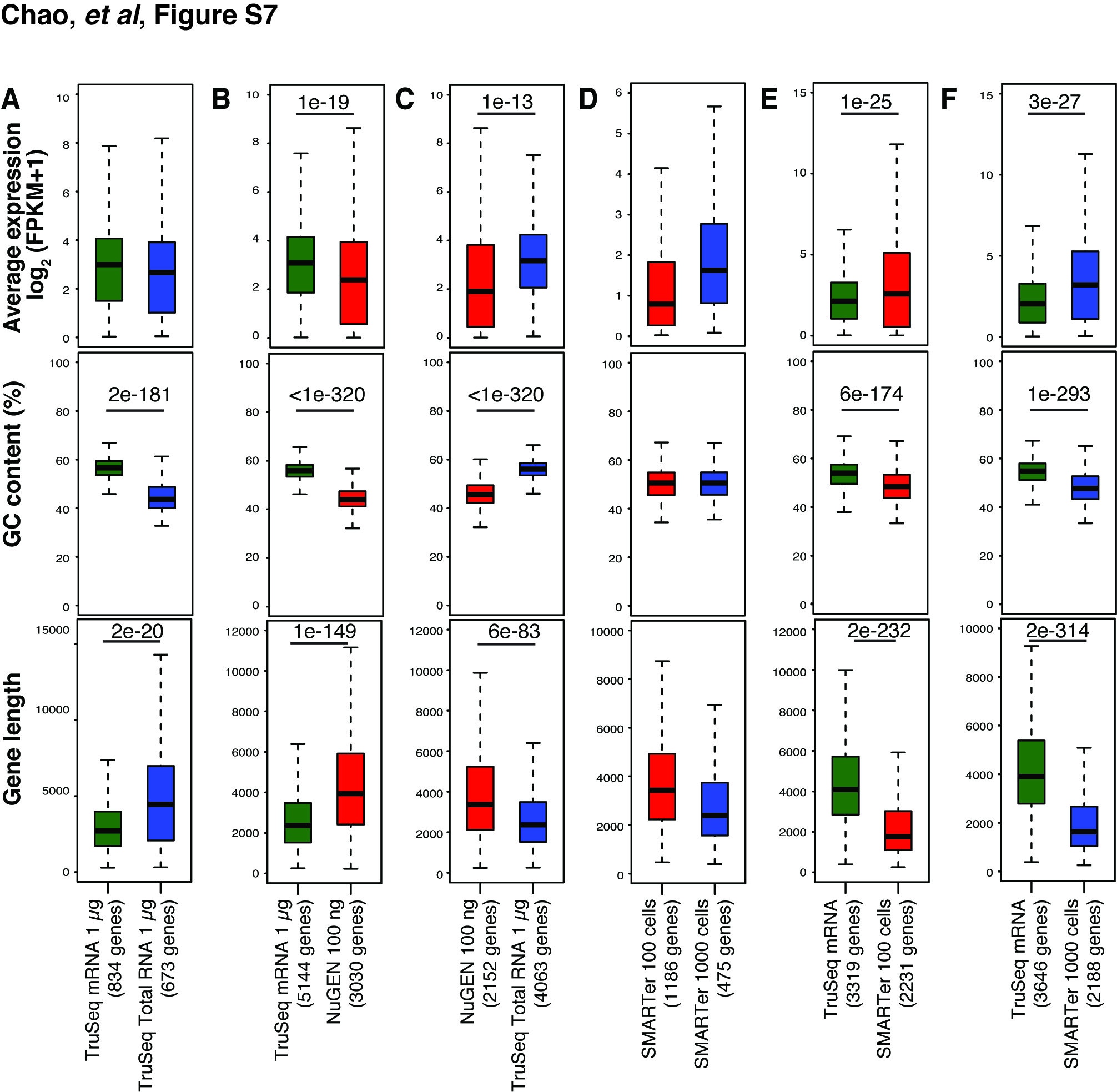

Supplement: Supplementary file 7 — Figure S7. Box plots of gene expression, GC content and gene length for the genes with elevated expression estimation in one protocol compared to the other protocol. Top figures are box plots of gene expression in log2(FPKM+ 1). Middle figures are box plots of GC content. Bottom figures are box plots of gene length. Panels A-C are for the standard input methods. Panels D-F are for the SMARTer Ultra Low RNA Kit. Panel A shows TruSeq mRNA protocol vs. the TruSeq Total RNA protocol. Panel B shows the TruSeq mRNA protocol vs. the modified NuGEN protocol. Panel C shows the NuGEN protocol vs. the TruSeq Total RNA protocol. Panel D shows the SMARTer Ultra Low RNA Kit 100 cells vs. 1000 cells. Panel E shows the TruSeq mRNA protocol vs. the SMARTer ultra-low protocol (100 cells). Panel F shows the TruSeq mRNA protocol vs. the SMARTer protocol (1000 cells). (TIF 2445 kb) [file 12864_2019_5953_MOESM7_ESM.tif]
